# Leveraging integrative toxicogenomic approach towards development of stressor-centric adverse outcome pathway networks for plastic additives

**DOI:** 10.1007/s00204-024-03825-z

**Published:** 2024-08-03

**Authors:** Ajaya Kumar Sahoo, Nikhil Chivukula, Shreyes Rajan Madgaonkar, Kundhanathan Ramesh, Shambanagouda Rudragouda Marigoudar, Krishna Venkatarama Sharma, Areejit Samal

**Affiliations:** 1 Computational Biology Group, The Institute of Mathematical Sciences (IMSc), CIT Campus, Taramani, Chennai, 600113 India; 2 Homi Bhabha National Institute (HBNI), Mumbai, 400094 India; 3 Ministry of Earth Sciences, National Centre for Coastal Research, Government of India, Pallikaranai, Chennai, 600100 India

**Keywords:** Plastic additives, Adverse outcome pathway (AOP), Toxicogenomics, Stressor–AOP network, Network analysis, Risk assessment

## Abstract

Plastics are widespread pollutants found in atmospheric, terrestrial and aquatic ecosystems due to their extensive usage and environmental persistence. Plastic additives, that are intentionally added to achieve specific functionality in plastics, leach into the environment upon plastic degradation and pose considerable risk to ecological and human health. Limited knowledge concerning the presence of plastic additives throughout plastic life cycle has hindered their effective regulation, thereby posing risks to product safety. In this study, we leveraged the adverse outcome pathway (AOP) framework to understand the mechanisms underlying plastic additives-induced toxicities. We first identified an exhaustive list of 6470 plastic additives from chemicals documented in plastics. Next, we leveraged heterogenous toxicogenomics and biological endpoints data from five exposome-relevant resources, and identified associations between 1287 plastic additives and 322 complete and high quality AOPs within AOP–Wiki. Based on these plastic additive–AOP associations, we constructed a stressor-centric AOP network, wherein the stressors are categorized into ten priority use sectors and AOPs are linked to 27 disease categories. We visualized the plastic additives–AOP network for each of the 1287 plastic additives and made them available in a dedicated website: https://cb.imsc.res.in/saopadditives/. Finally, we showed the utility of the constructed plastic additives–AOP network by identifying highly relevant AOPs associated with benzo[a]pyrene (B[a]P), bisphenol A (BPA), and bis(2-ethylhexyl) phthalate (DEHP) and thereafter, explored the associated toxicity pathways in humans and aquatic species. Overall, the constructed plastic additives–AOP network will assist regulatory risk assessment of plastic additives, thereby contributing towards a toxic-free circular economy for plastics.

**Supplementary Information:**

The online version contains supplementary material available at 10.1007/s00204-024-03825-z.

## Introduction

Plastics are the most widely produced synthetic chemicals, roughly constituting about 10% of solid waste generated globally (Thompson et al. 2009; Geyer et al. 2017). Extensive usage followed by improper waste management of plastics have made them ubiquitous pollutants in atmosphere, terrestrial and aquatic environments (Parthasarathy et al. 2019; Li et al. 2021b; Hecker et al. 2023). Plastics comprise various chemicals including polymers, solvents, additives and unintentional chemical residues resulting from the manufacturing process (Geyer et al. 2017). In particular, additives are chemicals that are intentionally added during the plastic manufacturing process to achieve specific desirable properties such as flexibility, reduced flammability, pigmentation, and make up nearly 50% by weight of the plastics (Hahladakis et al. 2018; UNEP 2023; Maes et al. 2023). These plastic additives are not covalently bonded to plastic, and thus can be potentially released into the environment throughout the plastic life cycle (Hermabessiere et al. 2017; UNEP 2023; Maddela et al. 2023). Environmental exposure to such plastic additives has been observed to elicit various adverse health effects such as cancer, developmental defects, endocrine disruptions, and metabolic disruptions in humans and other species alike (Oehlmann et al. 2008; Meeker et al. 2009; Sendra et al. 2021; Maddela et al. 2023; Stevens et al. 2024), but the lack of information on their presence throughout the plastics life cycle hampered their risk assessment and eventually the product safety (UNEP 2023). In addition, these plastic additives can persist in the environment, bioaccumulate in various organisms, and have long-lasting ecological impacts (Herrera et al. 2022; Costa et al. 2023). Therefore, it is imperative to identify these plastic additives and perform their risk assessment to achieve a toxic-free circular economy for plastics.

In an attempt to improve and accelerate chemical toxicity testing in twenty-first century, the US National Research Council in their report titled ‘Toxicity Testing in the 21^st^ century: a vision and strategy’ advocated the use of new approach methodologies like in vitro and in silico high-throughput screening strategies (National Research Council 2007). In accordance with the report’s suggestions, Ankley et al. (2010) proposed the conceptual toxicological framework namely, adverse outcome pathway (AOP), that sequentially organizes the stressor-induced biological response as Key Events (KEs) across various levels of biological organizations, thereby capturing the mechanism of stressor-induced toxicity (Ankley et al. 2010; Villeneuve et al. 2014a). In an AOP, the originating KE corresponds to the stressor-induced molecular event and is termed molecular initiating event (MIE), whereas the terminal KE corresponds to the stressor-induced adverse effect and is termed adverse outcome (AO) (Ankley et al. 2010; Villeneuve et al. 2014a; OECD 2017). The sequential information in an AOP is represented in a linear setup where the flow of information is captured by directional links connecting different KEs (including MIEs and AOs), which are termed Key Event Relationships (KERs) (Villeneuve et al. 2014a, b; Vinken et al. 2017). Therefore, exploring toxicities induced by plastic additives using an AOP framework can streamline their risk assessment.

AOP–Wiki (https://aopwiki.org/) is the largest open-source repository which catalogs AOPs that are developed globally. In AOP–Wiki, an AOP is developed in a stressor–agnostic manner, where it captures a single toxicity pathway by linking a molecular perturbation (MIE) to an adverse effect (AO) of regulatory relevance (Leist et al. 2017; Knapen et al. 2018; Villeneuve et al. 2018). Thus, linking a stressor to different AOPs within AOP–Wiki can help identify all possible toxicity pathways associated with that stressor (Knapen et al. 2018).

Recently, we had leveraged heterogenous biological data from five exposome-relevant resources namely, ToxCast (Dix et al. 2007), Comparative Toxicogenomics Database (CTD) (Davis et al. 2023), DEDuCT (Karthikeyan et al. 2019, 2021), NeurotoxKb (Ravichandran et al. 2021b), and AOP–Wiki to extensively explore various toxicity pathways associated with inorganic cadmium-induced toxicity (Sahoo et al. 2024). Previously, (Aguayo-Orozco et al. 2019) had utilized biological endpoint data of chemicals screened through several high throughout toxicity assays in ToxCast to construct stressor–AOP network linking these chemicals to several developed AOPs within AOP–Wiki. Such a construction enabled exploration of the adverse effects associated with this chemical space from a mechanistic perspective (Aguayo-Orozco et al. 2019). Therefore, a data integrative approach can help identify AOPs within AOP–Wiki that are relevant for plastic additives-induced toxicity. Such plastic additive–AOP associations can aid in the development of stressor-centric AOP network for plastic additives that will provide a holistic view of plastic additives-induced adverse effects, thereby aiding in its regulatory decision-making.

In this study, we relied on the United Nations (UN) report titled ‘Chemicals in Plastics—A Technical Report’ (UNEP 2023) that cataloged chemicals found in plastics from two independent studies by Aurisano et al. (2021b) and Wiesinger et al. (2021) and identified plastic additives among them based on their reported functions. First, we relied on AOP–Wiki, and systematically curated complete and connected high quality AOPs. Next, we leveraged the toxicogenomics and biological endpoints data from ToxCast, CTD, DEDuCT, NeurotoxKb, and AOP–Wiki to identify KEs associated with the curated plastic additives. Thereafter, based on these associated KEs, we constructed stressor-centric AOP network for plastic additives (designated as plastic additives–AOP network). Furthermore, we linked the plastic additives to their corresponding priority use sectors and the AOPs to their corresponding disease categories in the constructed plastic additives–AOP network. Finally, we showed the utility of the constructed plastic additives–AOP network by identifying highly relevant plastic additive associated AOPs and leveraged published experimental evidence to explore plastic additive-induced toxicities including ecotoxicity. In sum, this is the first study leveraging heterogenous biological data to construct a large-scale stressor-centric AOP network for plastic additives.

## Materials and methods

### Compilation and curation of plastic additives

**Fig. 1:**
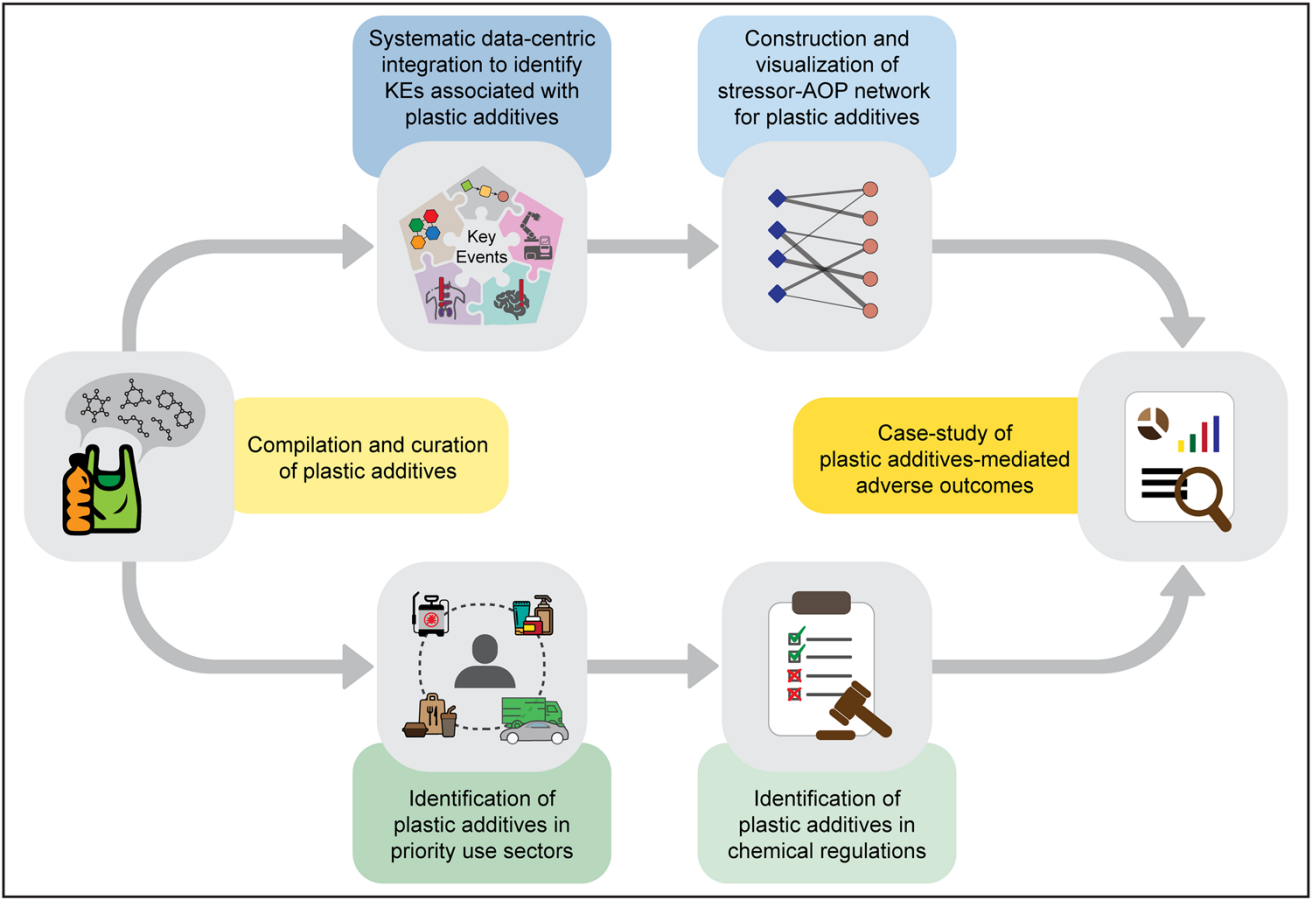
Summary of the workflow followed to identify plastic additives from chemicals found in plastics, followed by the exploration of their toxicity pathways through the construction of stressor-centric adverse outcome pathway (AOP) networks

Recently, the United Nations Environment Programme (UNEP) published a report titled ‘Chemicals in Plastics—A Technical Report’ (UNEP 2023) that provides an annex cataloging over 13,000 chemicals found in plastics and plastic manufacturing processes that were systematically curated by Aurisano et al. (2021b) and Wiesinger et al. (2021). Among the various chemicals in plastics, the compounds termed plastic additives define the desirable properties in the final plastic product (UNEP 2023). Plastic additives are intentionally added, constituting anywhere between 4% and 50% by weight in plastics (UNEP 2023), and can potentially leach into the environment as they are not covalently bonded to the plastic polymers, thus posing a risk to human health and environment (Maddela et al. 2023). Here, we relied on the annex provided by the UNEP report to identify the different plastic additives (Fig. 1).

We first compiled the chemicals and their corresponding chemical abstracts service (CAS) registry numbers provided by the UNEP report. Thereafter, we relied on the CAS common chemistry web portal (https://commonchemistry.cas.org/) to identify synonymous CAS registry numbers and mapped them to their latest identifiers to remove redundancy and duplications in chemical identifiers listed in the UNEP report. In case the CAS identifier provided by the UNEP report is not present in the portal, we used the identifier provided by the UNEP report, and finally compiled 13,640 unique chemicals.

Next, we observed that various terms were used in the UNEP report to identify functions of different chemicals in plastics. Notably, some of these chemicals were annotated only as non-intentionally added substances (NIAS), which we excluded from our analysis. Thereafter, we standardized the vocabulary of the associated functions by relying on various published sources (Koleske et al. 2011; Pritchard 2012; Al-Malaika et al. 2017; Coleman 2017; Geyer et al. 2017; Hahladakis et al. 2018; UNEP 2023). To identify the functions associated with plastic additives, we relied on several published sources and documents (Koleske et al. 2011; Pritchard 2012; BASF 2013; Al-Malaika et al. 2017; Coleman 2017; Geyer et al. 2017; Hahladakis et al. 2018; De Frond et al. 2021; UNEP 2023). The details of the curated functions associated with plastic additives, and their descriptions is provided in Table S1. We consider a chemical as a plastic additive if it has at least one annotated function that is associated with plastic additives. Through this extensive manual effort, we finally curated a list of 6470 plastic additives (Table S2) from the chemicals compiled in the UNEP report, and leveraged them for further analysis. Figure S1 illustrates the steps taken to curate a list of 6470 plastic additives from chemicals documented to be found in plastics.

### Compilation of AOPs within AOP–Wiki

The AOP–Wiki (https://aopwiki.org/) is the largest publicly accessible repository, hosted by the Society for the Advancement of Adverse Outcome Pathways (SAAOP), which compiles and organizes various AOPs developed globally. An AOP is a linear framework that captures information on biological targets (called Molecular Initiating Events or MIEs) perturbed due to the action of an external stressor, and the cascade of events (called Key Events or KEs) following this perturbation that culminates into Adverse Outcomes (AOs) (Ankley et al. 2010; OECD 2016, 2017). AOP–Wiki hosts this information on several AOPs documented in the form of KEs and the relationship between these KEs, known as Key Event Relationships (KERs), both of which are supported by scientific evidence.

To access the latest information available within AOP–Wiki, we downloaded the XML file (released on 1 January 2024) from ‘Project Downloads’ page in AOP–Wiki. Then, we utilized an in-house python script to parse the XML file and extract various information associated with AOPs like AOP identifier, AOP title, associated KEs (including MIEs and AOs) and KERs, linked stressors, status according to OECD and SAAOP, and biological applicability information such as taxonomy, sex and life-stage of the organism, and their corresponding weight of evidence. In addition, we also extracted information associated with KEs, such as KE title, KE identifier, level of biological organization, action name, object name, object identifiers and process name, and information associated with KERs like upstream/downstream KEs, evidence for biological plausibility of KER, adjacency, and the extent of quantitative understanding of KER.

### Identification of ‘high confidence AOPs’ within AOP–Wiki

Within AOP–Wiki, the AOPs are continuously updated based on current understanding and availability of novel experimental data, and thus, the AOPs are living documents (https://aopwiki.org/handbooks/4). Therefore, we relied on a systematic workflow developed in our previous work (Sahoo et al. 2024), to filter high quality and complete AOPs within AOP–Wiki (Fig. S2). First, we filtered out AOPs with SAAOP status as ‘archived’. Next, we manually checked and removed AOPs that have KE title as ‘unknown’ or lacked any KEs or KERs (Fig. S2). Next, we checked for the presence of disconnected components in AOPs using NetworkX library (Hagberg et al. 2008) in python, and manually updated and filtered out AOPs that contained disconnected components. Finally, we checked the remaining AOPs for presence of MIEs, AOs and a directed path between MIE and AO, and filtered out AOPs that did not contain any such path (Fig. S2). This combined computational and manual effort led to the identification of 328 complete, connected and high quality AOPs within AOP–Wiki (last accessed on 15 February 2024) which we designate as ‘high confidence AOPs’ (Fig. S2; Table S3). The 328 high confidence AOPs comprise 1107 unique KEs (Table S4) and 1717 unique KERs (Table S5).

### Identification of KEs associated with plastic additives

Based on our previous work (Sahoo et al. 2024), we relied on a systematic and comprehensive data-centric integration method to identify the KEs within AOP–Wiki that are associated with plastic additives by utilizing toxicogenomics and biological endpoints data from five exposome-relevant resources: ToxCast (Dix et al. 2007), Comparative Toxicogenomics Database (CTD) (Davis et al. 2023) (https://ctdbase.org/), DEDuCT (Karthikeyan et al. 2019, 2021) (https://cb.imsc.res.in/deduct/), NeurotoxKb (Ravichandran et al. 2021b) (https://cb.imsc.res.in/neurotoxkb/) and AOP–Wiki (https://aopwiki.org/).

#### Using ToxCast

US EPA’s ToxCast program provides high throughput in vitro bioactivity assay data for thousands of chemicals tested across several assays (Dix et al. 2007). Importantly, the ToxCast data include assay annotations and information on associated bioprocess and genes that can aid in the identification of KEs (specifically MIEs) associated with the corresponding active chemical (Knapen et al. 2018; Aguayo-Orozco et al. 2019; Jeong and Choi 2020; Sahoo et al. 2024). First, we downloaded the latest ToxCast invitrodb version 4.1 (EPA US 2023) dataset from the US EPA repository (https://www.epa.gov/chemical-research/exploring-toxcast-data). Next, we retrieved chemicals and their corresponding assay endpoints from the ‘mc5-6_winning_model_fits-flags_invitrodb_v4_1_SEPT2023.csv’ file and filtered chemicals with active assay endpoints (‘hitc’ ≥ 0.9) (Feshuk et al. 2023). Furthermore, we retrieved the ‘activatory’ or ‘inhibitory’ response of these active chemicals by relying on the ‘top’ value of the corresponding winning model from the ‘mc4_all_model_fits_invitrodb_v4_1_SEPT2023.csv’ file (Feshuk et al. 2023).

Sometimes, chemicals exhibit their activity in a narrow range of concentrations that coincides with that of cell stress and cytotoxicity, thereby leading to non-specific activation of reporter genes. Such phenomena are termed ‘cytotoxicity-associated bursts’ and can lead to inaccurate assay endpoint readings (Judson et al. 2016). Therefore, in this study, we identified such cytotoxicity-associated bursts for plastic additives tested within ToxCast, and did not consider those endpoints for mapping with KEs within AOP–Wiki (Fig. S3).

To identify cytotoxicity-associated bursts within ToxCast, Judson et al*.* (Judson et al. 2016) proposed the following Z-score metric:

$$Z\left( {chemical,\,assay} \right) = \frac{{ - logAC_{50} \left( {chemical, assay} \right) - median\left[ { - logAC_{50} \left( {chemical,cytotox} \right)} \right]}}{{\text{global cytotoxicity MAD}}}$$

wherein ‘logAC_50_(chemical,assay)’ is the logarithm of the AC_50_ value of the chemical in the assay, ‘logAC_50_(chemical,cytotox)’ is the logarithm of the AC_50_ value of the chemical in the corresponding cytotoxicity assay and the ‘global cytotoxicity MAD’ is the median of the MAD (median average deviations) of the logAC_50_(chemical,cytotox) distributions across all chemicals (Judson et al. 2016). For a given chemical, assays having Z-score values lying between + 3 and -3 were considered as cytotoxicity-associated bursts (Judson et al. 2016). Here, we relied on this Z-score metric by Judson et al*.* to identify cytotoxicity-associated bursts corresponding to the plastic additives tested within ToxCast.

The ‘cytotox_invitrodb_v4_1_SEPT2023.xlsx’ file in ToxCast invitrodb version 4.1 provides the global cytotoxicity MAD and logAC_50_(chemical, cytotox) (‘cytotox_median_log’) for chemicals across various cytotoxicity assays (Feshuk et al. 2023). We retrieved these values for the plastic additives tested within ToxCast, computed their Z-scores and discarded assays that had a Z-score value lying between + 3 and − 3. Through this process, we identified 1108 assay endpoints associated with 1327 plastic additives, and proceeded to map them to KEs within AOP–Wiki (Fig. S3).

First, we retrieved the genes associated with these 1108 assay endpoints from the ‘assay_annotations_invitrodb_v4_1_SEPT2023.xlsx’ file and the details of assay endpoint-gene mappings from ‘assay_gene_mappings_invitrodb_v4_1_SEPT2023’ file. Next, we leveraged KE-gene annotations provided by Saarimäki et al. (2023) to identify gene sets associated with KEs having biological level of organization as either molecular or cellular. Thereafter, we mapped these KEs to ToxCast assay endpoints based on gene overlaps, and manually filtered the mappings based on the assay endpoint descriptions. Through this extensive toxicogenomics based manual curation, we obtained 212 assay endpoints mapped to 115 KEs for 1129 of the 6470 curated plastic additives (Fig. S3).

#### Using CTD

Comparative Toxicogenomics Database (CTD) (Davis et al. 2023) (https://ctdbase.org/) is one of the largest toxicogenomics resources that compiles data on chemical–gene/protein, chemical–phenotype, chemical–disease, and gene–disease associations from published literature. The concept of chemical (C), gene (G), phenotype (P) and disease (D) tetramers, i.e. CGPD–tetramers, was proposed to understand the phenotypes and diseases that result from the interaction of chemicals with genes (Davis et al. 2020; Jeong et al. 2023). Based on our previous work (Sahoo et al. 2024), we retrieved the CGPD–tetramers associated with plastic additives within CTD, and leveraged them to identify the associated KEs within AOP–Wiki.

First, we downloaded the CTD’s January 2024 release and constructed the CGPD–tetramers for the plastic additives based on the workflow proposed in our previous work (Sahoo et al. 2024). This resulted in the identification of 124,496 tetramers comprising 258 chemicals, 2932 genes, 1489 phenotypes and 690 diseases (Table S6). Furthermore, we generated the immediate neighbor GO terms for the CGPD–tetramer phenotype GO terms using the GOSim package (Fröhlich et al. 2007) available in R programming language. Thereafter, we overlapped the GO terms with the process identifiers of KEs within AOP–Wiki, and manually inspected to identify 307 KEs associated with 266 phenotypes for 241 of the 6470 curated plastic additives (Table S7). We also manually inspected the disease terms to identify 157 KEs associated with 315 diseases for 232 of the 6470 curated plastic additives (Table S7).

#### Using DEDuCT and NeurotoxKb

DEDuCT (Karthikeyan et al. 2019, 2021) (https://cb.imsc.res.in/deduct/) is one of the largest databases that compiles curated information on endocrine disrupting chemicals (EDCs) and their corresponding endocrine-mediated endpoints from published literature. Therefore, we compiled the endocrine-mediated endpoints corresponding to plastic additives within DEDuCT, and considered them to find associated KEs within AOP–Wiki. We manually inspected the endpoints and titles of KEs within AOP–Wiki, and identified 165 KEs that are associated with 188 endocrine-mediated endpoints for 203 of the 6470 curated plastic additives (Table S7).

NeurotoxKb (Ravichandran et al. 2021b) (https://cb.imsc.res.in/neurotoxkb/) is a manually curated resource on mammalian neurotoxicity associated endpoints of environmental chemicals curated from published literature. Therefore, we compiled the neurotoxic endpoints corresponding to plastic additives within NeurotoxKb, and considered them to find associated KEs within AOP–Wiki. We manually inspected the neurotoxic endpoints and KEs within AOP–Wiki, and identified 25 KEs that are associated with 24 neurotoxic endpoints for 92 of the 6470 curated plastic additives (Table S7).

#### Using AOP–Wiki

AOP–Wiki also catalogs the stressor information for each AOP, where there exists well documented evidence of such stressor(s) showing response at multiple KEs, including MIEs (https://aopwiki.org/handbooks/4). Therefore, we relied on the stressor information within AOP–Wiki to identify KEs associated with plastic additives. We retrieved information on stressors associated with each AOP, and identified 33 AOPs to be associated with 42 of the 6470 curated plastic additives (Table S7). Thereafter, we identified 178 KEs in these 33 AOPs that are associated with plastic additives.

Overall, we identified 688 KEs that are associated with 1314 plastic additives (out of the 6470 plastic additives in our curated list) through the integration of heterogenous toxicogenomics and biological endpoints data from five exposome-relevant resources: ToxCast, CTD, DEDuCT, NeurotoxKb and AOP–Wiki (Table S7).

### Compilation of chemical lists for priority use sectors of plastic additives

Globally, plastics are used across different scales and for various applications. The UNEP report (UNEP 2023) has identified ten priority use sectors, based on the likelihood of exposure of chemicals in plastic products in these sectors to humans and environment. The ten priority use sectors include ‘Toys and other children’s products’, ‘Furniture’, ‘Packaging including food contact materials’, ‘Electrical and electronic equipment’, ‘Transport’, ‘Personal care and household products’, ‘Medical devices’, ‘Building materials’, ‘Synthetic textiles’, and ‘Agriculture, aquaculture and fisheries’. To identify the plastic additives being used in each of the priority use sectors, we first compiled the list of chemicals in use in each of these sectors.

Chemical and Products Database (CPDat) (Dionisio et al. 2018) (https://www.epa.gov/chemical-research/chemical-and-products-database-cpdat) is among the largest resources that catalogs the presence of chemicals in various consumer products. For each product, CPDat assigns a Product Use Category (PUC) based on the general category and product type mentioned in the original data source (Dionisio et al. 2018). Here, we relied on CPDat to identify the chemicals in use in each of the ten priority use sectors. We accessed the CPDat data file (Williams 2021) (last accessed on 15 February 2024) to compile the list of chemicals associated with different PUCs, and identified 20 PUCs to be grouped under the different priority use sectors (Fig. S4; Table S8).

The CompTox Chemicals Dashboard (Williams et al. 2017, 2021) (https://comptox.epa.gov/dashboard/) is one of the largest public repositories that provides access to different lists of chemicals associated with projects, publications, source databases or collections (https://comptox.epa.gov/dashboard/chemical-lists). Here, we queried the chemical lists based on their description, and identified chemical lists associated with the different priority use sectors (Fig. S4; Table S8). The chemical lists from CompTox which were used included food contact chemicals (Qian et al. 2018; Groh et al. 2021), chemicals associated with plastic packaging (Groh et al. 2019), chemicals associated with pesticides (Lewis et al. 2016; Menger et al. 2021), and chemicals associated with plastic toys (Aurisano et al. 2021a). Furthermore, we compiled chemicals from an in-house repository namely, Fragrance Chemicals in Children’s Products (FCCP) (Ravichandran et al. 2022a) (https://cb.imsc.res.in/fccp) as chemicals found in the use sector ‘Toys and other children’s products’. Finally, we compiled the chemicals in use in each of the priority use sectors, and identified plastic additives present in each sector (Table S8).

### Construction and visualization of the stressor–AOP network

Stressor–AOP network provides a holistic view of chemical perturbances across different AOPs (Aguayo-Orozco et al. 2019). To better understand the perturbances caused by the different plastic additives, we constructed a stressor–AOP network as a bipartite graph that linked various plastic additives to different AOPs within AOP–Wiki. To obtain high confidence associations between plastic additives and AOPs, we relied only on the curated list of 328 high confidence AOPs (Table S3).

To obtain the stressor–AOP network for plastic additives, we initially linked plastic additives to AOPs if they share at least one associated KE, and thereafter, characterized each link between a stressor and an AOP based on the coverage score and level of relevance. Coverage score of a stressor–AOP link is defined as the ratio of number of KEs within that AOP associated with the stressor to the total number of KEs within that AOP (Chai et al. 2021). Coverage score is a real number that takes a value between 0 and 1 and we denote this score as the edge weight of linkage between a stressor and an AOP in our stressor–AOP network. Next, we realized that the plastic additives were associated with different AOPs with varying levels of relevance. Therefore, we propose the following five-level criterion to qualitatively understand the relevance of associations:

- *Level 1*: The stressor is associated with at least one KE within an AOP, where the KE is neither MIE nor AO within that AOP.
- *Level 2*: The stressor is associated with at least one AO within an AOP, but not associated with any MIE within that AOP.
- *Level 3*: The stressor is associated with at least one MIE within an AOP, but not associated with any AO within that AOP.
- *Level 4*: The stressor is associated with at least one MIE and one AO within an AOP.
- *Level 5*: The stressor is associated with at least one MIE and one AO within an AOP and there exists a directed path between the associated MIE and AO.

Table S9 contains all the data on the stressor–AOP network constructed for plastic additives, including the coverage score and level of relevance for each of the stressor–AOP links. We visualized this stressor–AOP network of plastic aditives using Cytoscape (Shannon et al. 2003).

## Results and discussion

### Exploration of the curated list of plastic additives

Plastic additives are chemicals that are added to plastics to achieve specific desirable properties in the end product (Pritchard 2012; Hahladakis et al. 2018). External stress on such products can cause the separation of these additives, thereby leading to their release into the environment and eventually posing risks to humans and ecosystems (Maddela et al. 2023). In this study, we curated a list of plastic additives from chemicals cataloged in the UNEP report titled ‘Chemicals in Plastics—A Technical Report’ (Table S2) (UNEP 2023) and explored their potential risks by systematically integrating the associated heterogenous biological endpoints within the context of adverse outcome pathway (AOP) framework (Fig. 1).

The UNEP report provides functional annotations for each of these chemicals based on two independent studies by Aurisano et al. (2021b) and Wiesinger et al. (2021). Notably, Wiesinger et al*.* observed that their text mining approach for identifying these functional annotations lacked context sensitivity, leading to some inaccuracies. Despite this limitation, the UNEP report remains the most comprehensive source cataloging chemicals found in plastics and their associated functions. Therefore, we relied on the functional annotations provided by the report to identify 6470 chemicals with reported functions, which we designate as 'plastic additives' in this study (Methods; Table S2).

Among the 6470 plastic additives, we observed that many chemicals (3217 of 6470) provide a variety of functions to the plastics, with colorants being the most frequently associated function (3675 of 6470) (Methods; Table S2). Furthermore, we observed that majority of plastic additives (4309 of 6470) are found in products made by different priority use sectors, of which 3963 additives are found in the use sector ‘Packaging, including food contact materials’ (Methods; Table S2).

**Fig. 2:**
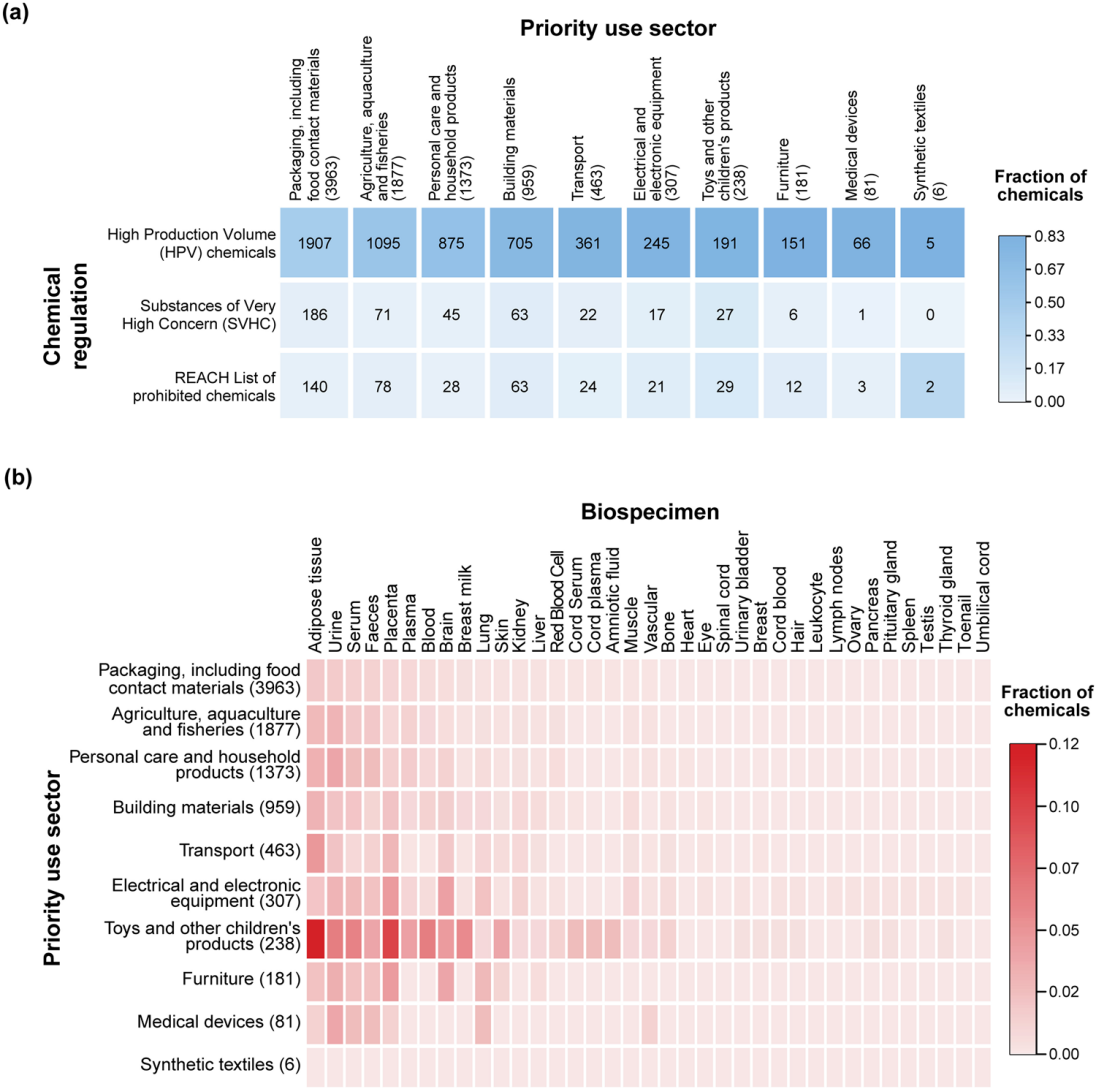
Identification of plastic additives in different chemical regulations and human biospecimens. **a** Heatmap depicting the presence of plastic additives from ten priority use sectors in chemical regulations. The number of the plastic additives from the priority use sector in each of the chemical regulations is denoted in the heatmap. **b** Heatmap depicting the presence of plastic additives from ten priority use sectors in different human biospecimens based on published exposure studies

Next, we relied on the United States high production volume (USHPV) chemical list (https://comptox.epa.gov/dashboard/chemical-lists/EPAHPV) and Organisation for Economic Co-operation and Development high production volume (OECD HPV) chemical list (https://hpvchemicals.oecd.org/ui/Search.aspx) and identified 2084 of 6470 plastic additives to be high production volume (HPV) chemicals (Table S2). Notably, among these HPV plastic additives, we found 154 additives to be known endocrine disrupting chemicals (EDCs) with experimental evidence for endocrine disruption in humans or rodents from DEDuCT (Karthikeyan et al. 2019, 2021), and 101 additives as potential carcinogens based on IARC monographs on identification of carcinogenic hazards to humans (https://monographs.iarc.who.int/list-of-classifications/) (Table S2). Furthermore, we observed that 215 additives are identified as substances of very high concern (SVHC) (https://echa.europa.eu/candidate-list-table) by European Chemicals Agency (ECHA) and 412 additives are prohibited for use as per REACH regulation (Table S2). Figure 2a shows the distribution of HPV, SVHC and REACH prohibited plastic additives across the ten priority use sectors.

### Plastic additives are accumulated in various human biospecimens

Humans are exposed to various plastic additives via direct contact, inhalation or ingestion, which can eventually accumulate in different human tissues and potentially lead to various adverse health effects (Meeker et al. 2009; UNEP 2023). To explore the plastic additives detected in various human biospecimens, we relied on two databases namely, Tissue-specific Exposome Atlas (TExAs) (Ravichandran et al. 2021a) (https://cb.imsc.res.in/texas/) and Exposome–Explorer (Neveu et al. 2020) (http://exposome-explorer.iarc.fr/) which have compiled the presence of environmental chemicals as xenobiotics in different human tissues from published exposure studies. Although, these two databases have compiled information from limited human exposure studies, they have documented 204 of the 6470 plastic additives to be accumulated as xenobiotics in 37 different human biospecimens (Fig. 2b; Table S2). Moreover, we observed that plastic additives from nine of the ten priority use sectors have been documented as xenobiotics in human biospecimens namely, faeces, serum, urine, lung, placenta, and adipose tissue (Fig. 2b). Note, the use sector ‘Synthetic textiles’ comprised the least number of additives (6 chemicals) in our curated list of 6470 additives, and there are no published exposure studies wherein their presence was detected in different human biospecimens.

### Stressor–AOP network for plastic additives

Stressor–AOP networks provide a panoramic visualization of the different AOPs associated with stressors of interest, and help in understanding the stressor-induced adverse biological effects (Aguayo-Orozco et al. 2019). In this study, we therefore constructed stressor–AOP network to understand the various adverse effects induced by plastic additives. First, we followed a systematic approach that involved data-centric integration of heterogenous toxicogenomics and biological endpoints data from five exposome-relevant resources namely, ToxCast, CTD, DEDuCT, NeurotoxKb and AOP–Wiki, and identified 688 KEs within AOP–Wiki to be associated with 1314 of the 6470 plastic additives (Methods; Table S7). Thereafter, we curated 328 high confidence AOPs within AOP–Wiki and mapped them to plastic additives if at least one KE within that AOP is associated with the plastic additive. Based on these plastic additive–AOP associations, we constructed a plastic additives-centric bipartite stressor–AOP network comprising two types of nodes namely, 1287 plastic additives and 322 high confidence AOPs, and 46,243 stressor–AOP links as edges between the two types of nodes, and we designate this bipartite network as plastic additives–AOP network. Notably, we observed that AOP–Wiki documented only 37 of the 1287 plastic additives in the constructed stressor–AOP network to be associated with 27 of the 322 high confidence AOPs in the network.

Next, we leveraged the KEs associated with plastic additives to compute the coverage score for the stressor–AOP links in the plastic additives–AOP network and observed that 20 plastic additives are associated with all the KEs (coverage score = 1.0) in 15 high confidence AOPs, and these stressor–AOP links were otherwise not documented in AOP–Wiki (Methods; Table S9). Moreover, we calculated the levels of relevance for the stressor–AOP links in the plastic additives–AOP network and observed that 27,189 links between 1155 plastic additives and 288 AOPs are classified as Level 1, 4236 links between 345 plastic additives and 241 AOPs are classified as Level 2, 14,187 links between 1152 plastic additives and 139 AOPs are classified as Level 3, and 631 links between 118 plastic additives and 98 AOPs are classified as Level 5 (Methods; Table S9). Note, the stressor–AOP links with Level 4 relevance were also satisfied by Level 5 criterion, and therefore, there are no stressor–AOP links with Level 4 relevance in the constructed network (Methods; Table S9).

Next, we relied on the standardized disease ontology provided in Disease Ontology (Schriml et al. 2022) database (https://disease-ontology.org/do) to classify the AOPs based on their adverse outcomes (AOs). Based on the standardized ontology, we classified 322 AOPs into 26 disease classes based on their AOs (Tables S9, S10). Note that 125 of the 322 AOPs could not be classified under any standardized ontology provided by Disease Ontology, and we therefore marked them as ‘unclassified’. Importantly, we observed that cancer is the most represented disease category comprising 40 of the 322 AOPs in the plastic additives–AOP network (Table S9). Finally, we have linked the plastic additives to their corresponding priority use sectors and the AOPs to their corresponding disease categories in the plastic additives–AOP network (Table S9).

**Fig. 3:**
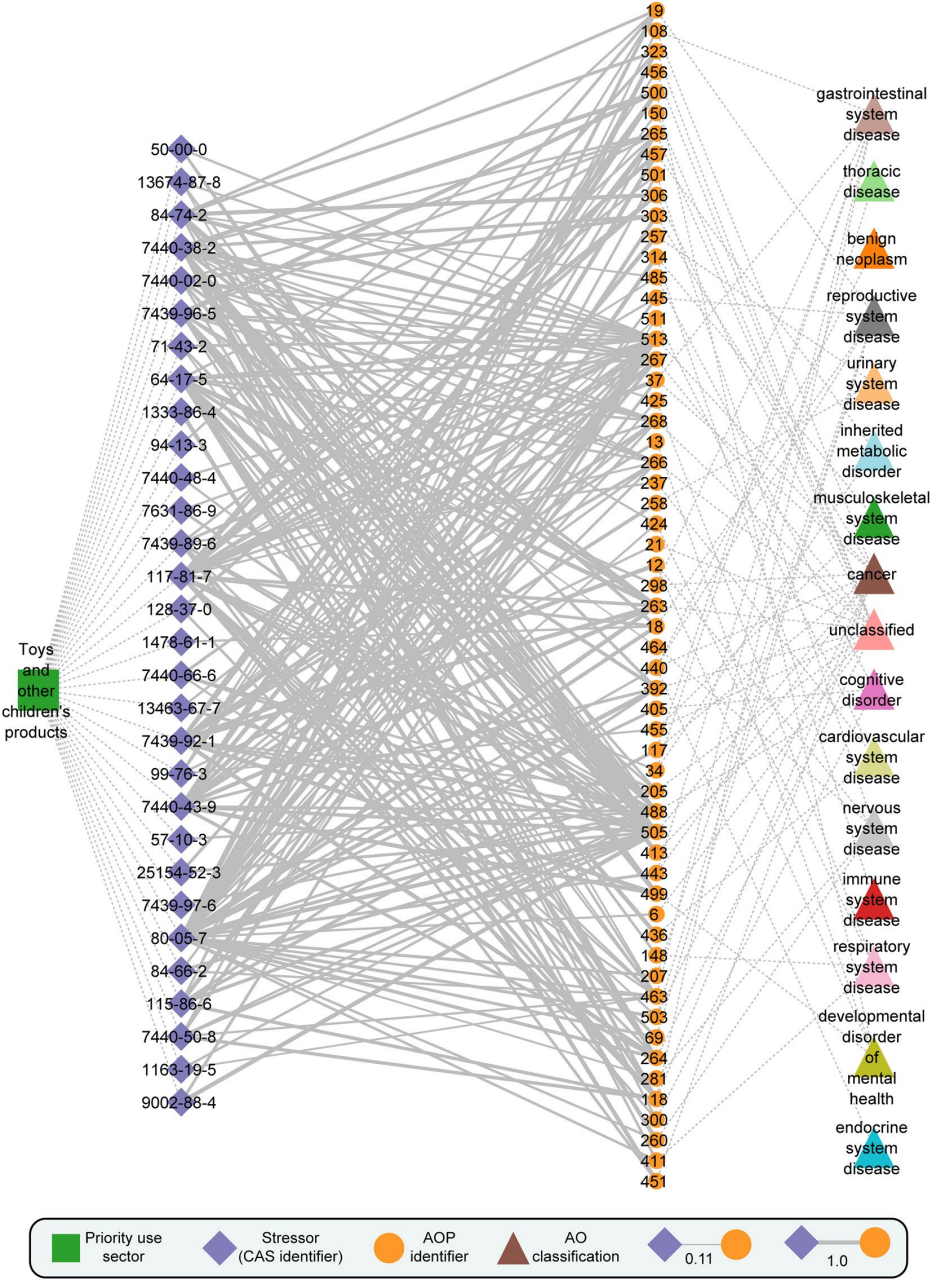
Visualization of a stressor-centric AOP network for plastic additives in the priority use sector ‘Toys and other children’s products’, along with the disease classifications of adverse outcomes (AOs) in AOPs. In the stressor–AOP network, only edges or stressor–AOP links with Level 5 relevance are shown. Furthermore, the edges or stressor–AOP links are weighted based on their coverage score, i.e., the fraction of KEs within AOP that are linked with the plastic additives

We relied on the graph visualization software Cytoscape (Shannon et al. 2003) to visualize the plastic additives–AOP network for each of the 1287 plastic additives, and make them available on a dedicated website: https://cb.imsc.res.in/saopadditives/. In the website, the plastic additives are grouped based on their priority use sectors. For instance, the priority use sector ‘Toys and other children’s products’ consists of 162 plastic additives, 301 AOPs and 8300 stressor–AOP links, wherein 4696 links between 148 plastic additives and 265 AOPs are classified as Level 1, 1460 links between 75 plastic additives and 170 AOPs are classified as Level 2, 1928 links between 144 plastic additives 117 AOPs are classified as Level 3, and 216 links between 30 plastic additives and 58 AOPs are classified as Level 5. Figure 3 shows a portion of the plastic additives–AOP network, comprising Level 5 stressor–AOP links for plastic additives in the use sector ‘Toys and other children’s products’.

**Fig. 4:**
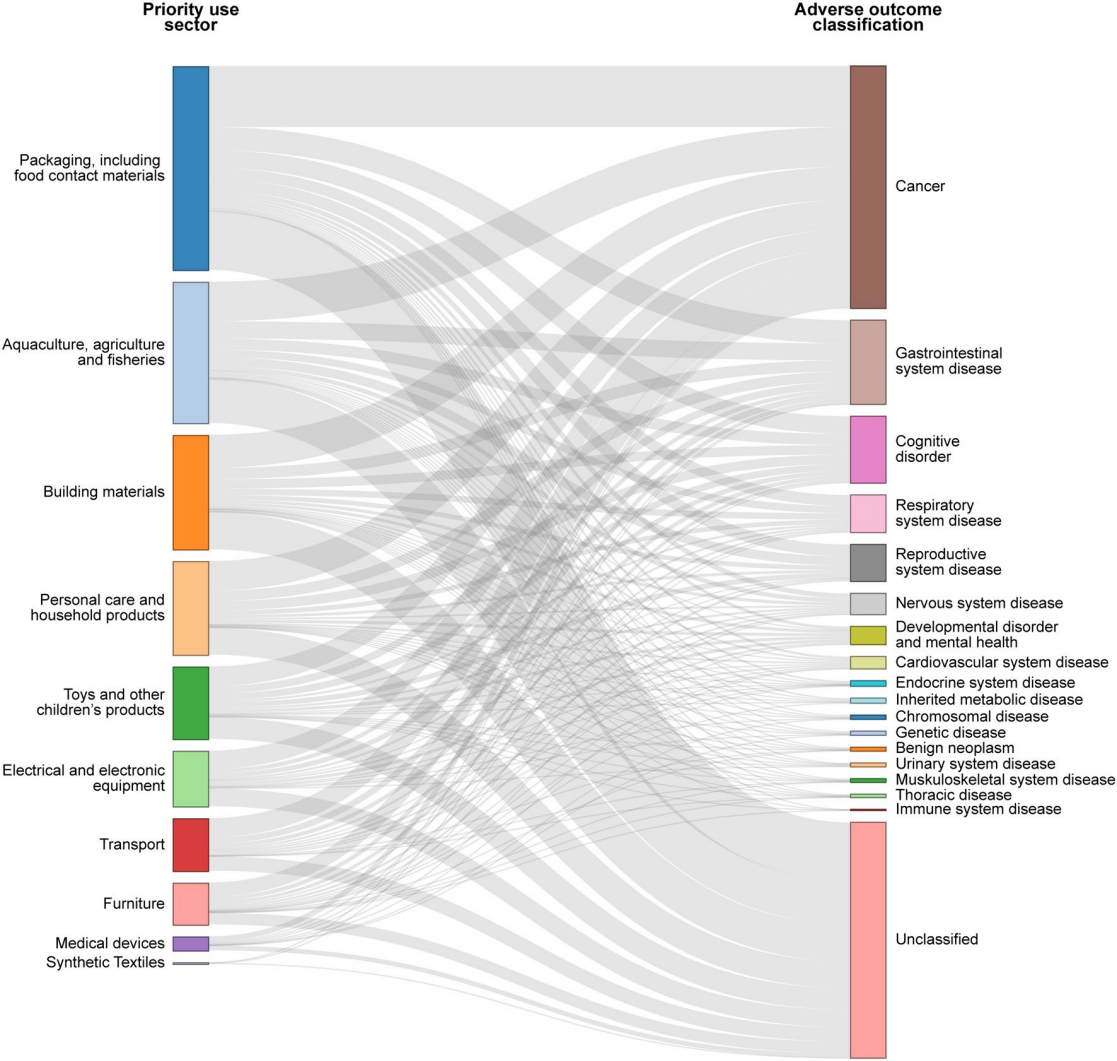
Aggregate visualization of the disease categories associated with AOPs in plastic additives stressor–AOP network with Level 5 relevance for each of the ten priority use sectors

In addition, the constructed plastic additives–AOP network can highlight the adverse outcomes induced by plastic additives across different use sectors. Figure 4 shows the disease categories linked with ten priority use sectors for plastic additives in the plastic additives–AOP network with Level 5 relevance, where cancer is the most represented disease category.

### Stressor–AOP network reveals highly relevant AOPs associated with plastic additives

A stressor–AOP network can help identify most relevant AOPs associated with each stressor which can further highlight the complexity and diversity among toxicity pathways induced by that stressor (Knapen et al. 2018). Here, we considered stressor–AOP links from the constructed plastic additives–AOP network with Level 5 relevance and coverage score threshold of 0.4, and identified 107 of the 1287 plastic additives to be associated with 88 of the 322 AOPs through 526 stressor–AOP links (Methods; Table S9). Note the coverage score threshold of 0.4 denotes that at least 40% of the KEs in that AOP are linked with the stressor (Chai et al. 2021; Sahoo et al. 2024). Notably, 15 of these 107 plastic additives are associated with more than 10 AOPs (Table 1). Among these 15 plastic additives, 14 are documented as EDCs in DEDuCT, and 10 are documented as carcinogens in IARC monographs (Table S2).

**Table 1:** Top 15 plastic additives based on the associated number of highly relevant AOPs in the plastic additives–AOP network with Level 5 stressor–AOP links

Plastic additive	Functions	Number of highly relevant stressor–AOP links	Number of stressor–AOP links not present in AOP–Wiki
Benzo[a]pyrene (CAS:50-32-8)	Plasticizers, Cross-linkers, Lubricants, Fillers	28	28
Bisphenol A (CAS:80-05-7)	Catalysts, Cross-linkers, Fillers, Antioxidants, Light stabilizers, Lubricants, Blowing agents, Plasticizers, Colorants, Flame retardants, Antistatic agents	27	26
Bis(2-ethylhexyl) phthalate (CAS:117-81-7)	Cross-linkers, Fillers, Light stabilizers, Fragrances, Plasticizers, Colorants	19	17
Arsenic (CAS:7440-38-2)	Biocides, Cross-linkers, Fillers, Colorants	16	13
Ethanol (CAS:64-17-5)	Biocides, Catalysts, Cross-linkers, Fillers, Light stabilizers, Lubricants, Fragrances, Colorants, Antistatic agents	15	13
Perfluorooctanoic Acid (CAS:335-67-1)	Biocides, Colorants	14	14
Triclosan (CAS:3380-34-5)	Biocides, Light stabilizers, Fragrances, Colorants	14	13
Cadmium (CAS:7440-43-9)	Biocides, Catalysts, Cross-linkers, Fillers, Heat stabilizers, Light stabilizers, Colorants, Pigments	14	11
Cadmium chloride (CAS:10108-64-2)	Biocides, Antioxidants, Heat stabilizers, Light stabilizers, Colorants, Flame retardants	13	12
Lead (CAS:7439-92-1)	Cross-linkers, Fillers, Antioxidants, Heat stabilizers, Light stabilizers, Lubricants, Other stabilizers, Colorants	13	10
Acrylamide (CAS:79-06-1)	Cross-linkers, Fillers, Colorants	13	13
Pentachlorophenol (CAS:87-86-5)	Biocides, Colorants	12	11
Manganese (CAS:7439-96-5)	Biocides, Catalysts, Fillers, Lubricants, Colorants	11	9
Oxygen (CAS:7782-44-7)	Blowing agents, Antioxidants	11	11
Lead acetate (CAS:301-04-2)	Cross-linkers, Flame retardants, Colorants	11	11

Among the AOPs associated with these 15 plastic additives, we observed that majority of the AOPs are identified through our systematic data integrative approach. Notably, we observed that AOP:263, AOP:264, AOP:265, AOP:267, and AOP:268 are shared among all 15 plastic additives (Table 1). Moreover, we observed that these five AOPs share the same MIE ‘Decrease, Coupling of oxidative phosphorylation’ (KE:1446) and AO ‘Decrease, Growth’ (KE:1521), while AOP:263 titled ‘Uncoupling of oxidative phosphorylation leading to growth inhibition via decreased cell proliferation’ is endorsed by Working Group of the National Coordinators of the Test Guidelines Programme (WNT) and the Working Party on Hazard Assessment (WPHA) under the OECD AOP development programme.

An AOP network constructed from stressor-specific AOPs can highlight interactions among the associated AOPs, thereby aiding in the assessment of stressor-induced toxicity (Pollesch et al. 2019; Rugard et al. 2020; Chai et al. 2021; Pogrmic-Majkic et al. 2022; Yang et al. 2022; Sahoo et al. 2024). Among the 15 plastic additives, we observed that benzo[a]pyrene (28 associated AOPs), bisphenol A (27 associated AOPs), and bis(2-ethylhexyl) phthalate (19 associated AOPs) are the top three chemicals based on the number of associated AOPs (Table 1). Although benzo[a]pyrene has been annotated as a plastic additive in this study, evidence suggests that it is more likely a contaminant or a byproduct resulting from the use of extender oils or carbon black in plastic production (Lassen et al. 2011; Alassali et al. 2020; Wiesinger et al. 2021). Nonetheless, all these three chemicals are well-known pollutants. Therefore, we constructed AOP networks for each of these chemicals and explored their potential human-relevant and ecotoxicology-relevant toxicity pathways.

### Exploration of toxicity pathways in AOP network constructed from benzo[a]pyrene-relevant AOPs

Benzo[a]pyrene (B[a]P or CAS:50-32-8) has the largest number of associated AOPs (28 AOPs), all of which were solely identified through our systematic data integrative approach (Table 1). These 28 AOPs are classified under various disease categories namely, cancer, gastrointestinal system disease, reproductive system disease, respiratory system disease, cognitive disorder, thoracic disease, and musculoskeletal system disease (Table S9). Previously, Yang et al. (2022) had constructed an AOP network for B[a]P-induced toxicity, but they relied only on CTD to identify KEs associated with B[a]P and focused only on B[a]P-induced male reproductive damages. Therefore, we relied on 28 AOPs associated with B[a]P-induced toxicity (which we designate as B[a]P–AOPs) and constructed an AOP network to explore various adverse effects associated with B[a]P.

**Fig. 5:**
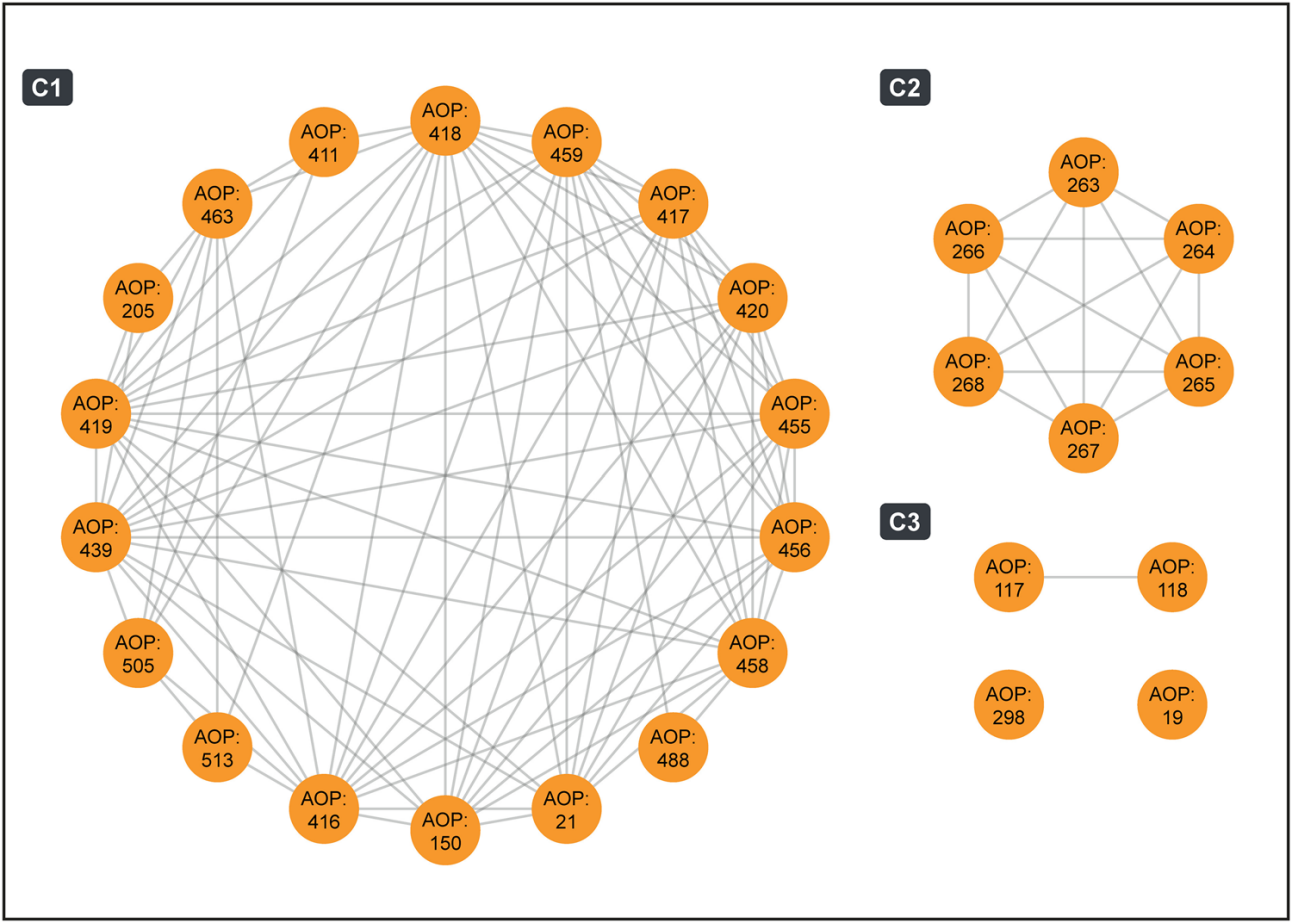
Undirected network of B[a]P–AOPs. Each node corresponds to B[a]P–AOP and an edge between two nodes denotes that the two AOPs share at least one KE. This undirected network has 3 connected components (with two or more nodes) which are labeled as C1, C2, and C3, and 2 isolated nodes

Next, we computed the cumulative weight of evidence (WoE) for each of these 28 B[a]P–AOPs based on their KER information to assess the biological plausibility (Ravichandran et al. 2022b; Sahoo et al. 2024). We observed that 9 of these 28 B[a]P–AOPs have ‘High’ cumulative WoE and 6 B[a]P–AOPs have ‘Moderate’ cumulative WoE (Table S11). Moreover, we observed that many of these 28 B[a]P–AOPs are applicable across various species and developmental stages (Table S11). Figure 5 shows the undirected AOP network representation of the 28 B[a]P–AOPs, where nodes represent B[a]P–AOPs and the edges represent the existence of shared KEs between two AOPs. We observed that the B[a]P–AOPs form three connected components (with two or more AOPs) and two isolated nodes, where the largest connected component (labeled C1) comprises 18 B[a]P–AOPs.

**Fig. 6:**
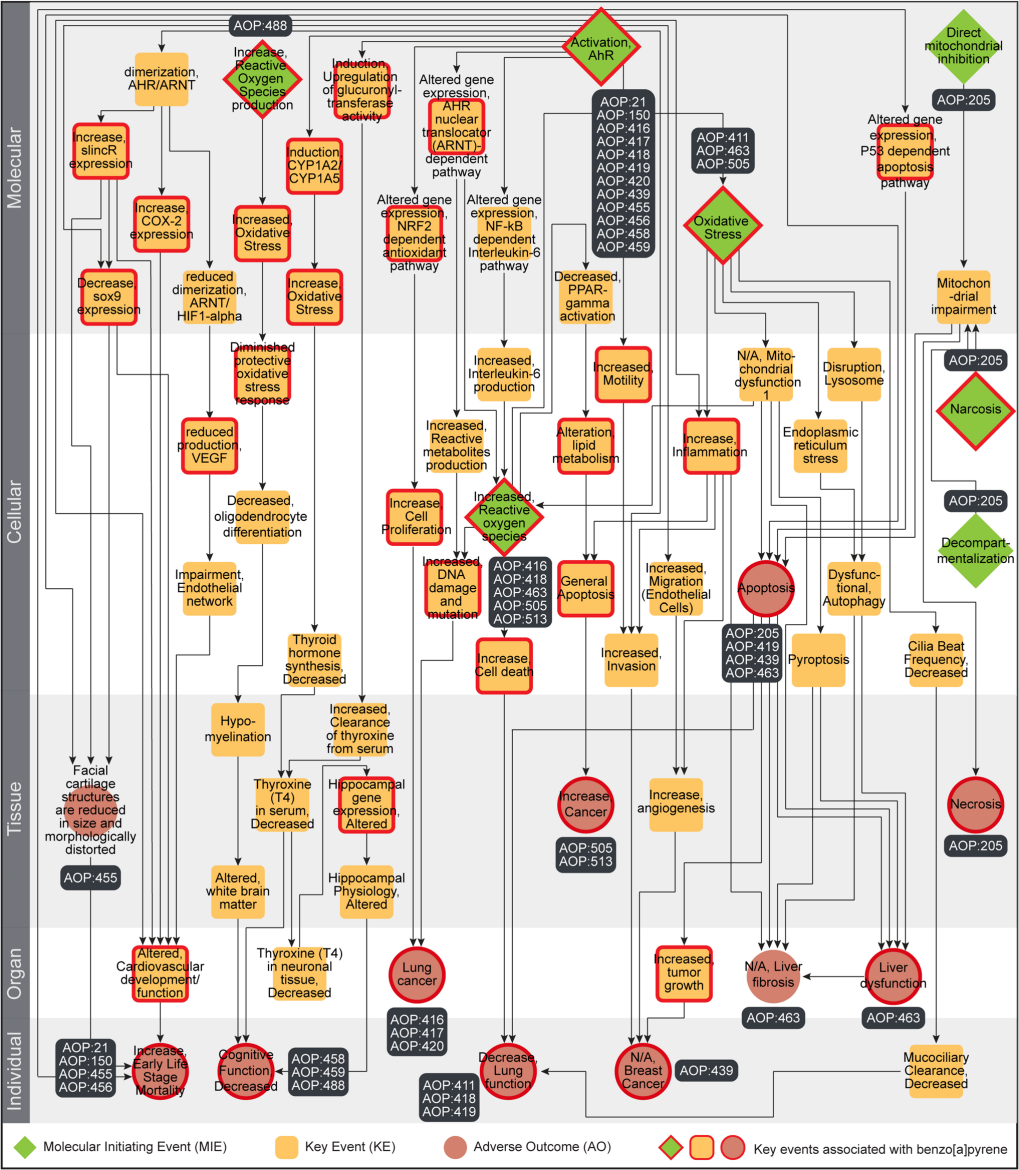
Directed network corresponding to the largest component in the undirected B[a]P–AOP network comprising 66 KEs and 99 KERs. Among the 66 KEs, 7 are categorized as MIEs (denoted as diamond), 11 are categorized as AOs (denoted as circle), and the remaining 48 are categorized as KEs (denoted as rounded square). The 36 KEs (including MIEs and AOs) associated with B[a]P are marked in ‘red’. In this figure, the 66 KEs are arranged vertically according to their level of biological organization (colour figure online)

We constructed and visualized a directed AOP network to explore the interactions among the B[a]P–AOPs present in the largest connected component C1 (Fig. 6). We observed that the directed network comprised 66 unique KEs (including 7 MIEs and 11 AOs) and 99 unique KERs (Fig. 6; Table S12). Among the 66 KEs, 36 KEs were associated with B[a]P-induced toxicity through our systematic data integrative approach, of which 5 are MIEs and 10 are AOs (Fig. 6). Notably, we observed that the toxicity pathway originating from MIE ‘Activation, AhR’ (KE:18), passing through KEs ‘Altered gene expression, NRF2 dependent antioxidant pathway’ (KE:1917) and ‘Increase, Cell Proliferation’ (KE:870), and eventually terminating at AO ‘Lung Cancer’ (KE:1670) consists of 4 of the 36 KEs associated with B[a]P-induced toxicity (Fig. 6). Upon further inspection, we identified that this toxicity pathway was captured in the AOP:420 titled ‘Aryl hydrocarbon receptor activation leading to lung cancer through sustained NRF2 toxicity pathway’, which was systematically built and supported by extensive literature survey and experimental data on B[a]P (Jin et al. 2022).

Next, we computed different node-centric network measures to explore various features of this directed network. We observed that the MIE ‘Activation, AhR’ (KE:18) has the highest out-degree of 16, while the KE ‘Altered, Cardiovascular development/function’ (KE:317) and AO ‘N/A, Liver fibrosis’ (KE:344) have the highest in-degree of 5 (Table S12). The MIE ‘Increased, Reactive oxygen species’ (KE:1115) has the highest betweenness centrality value, denoting that several toxicity pathways are passing through it in this network (Fig. S5) (Villeneuve et al. 2018). The KEs ‘Induction, CYP1A2/CYP1A5’ (KE:850) and ‘Altered gene expression, NF-kB dependent Interleukin-6 pathway’ (KE:1921), and MIEs ‘Activation, AhR’ (KE:18) and ‘Increase, Reactive Oxygen Species production’ (KE:257) have the highest eccentricity, denoting that they are the most remotely placed KEs in this network (Fig. S6) (Takes and Kosters 2011).

Finally, we relied on artificial intelligence (AI) based tool, AOP-helpFinder (Jornod et al. 2022; Jaylet et al. 2023) (https://aop-helpfinder.u-paris-sciences.fr/), and Abstract Sifter (Baker et al. 2017) (https://comptox.epa.gov/dashboard/chemical/pubmed-abstract-sifter/) to screen published literature and manually identified novel associations between the B[a]P-induced toxicities and the remaining 30 KEs in the directed AOP network (Table S13). In addition, we compiled auxiliary evidence for the 36 KEs that were associated with B[a]P-induced toxicity through our systematic data integrative approach. Furthermore, we compiled information on the type of evidence and the reported toxicity dosage values of B[a]P exposure from these published evidence (Table S13). To conclude, we performed two case studies to explore both the human-relevant and ecotoxicology-relevant B[a]P-induced toxicity pathways from this directed AOP network.

#### Toxicity pathway linking B[a]P exposure to liver fibrosis in humans

Liver fibrosis, which results from chronic damage to the liver, is a characteristic of many chronic liver diseases (Bataller and Brenner 2005). Previously, exposome-based studies had found a significant association between environmental chemicals such as B[a]P and different liver diseases (Cheung et al. 2020; Barouki et al. 2023). Here, we observed an emergent B[a]P-induced toxicity pathway originating from MIE ‘Activation, AhR’ (KE:18) and terminating at AO ‘N/A, Liver fibrosis’ (KE:344). Therefore, we relied on this emergent toxicity pathway to understand the rationale behind B[a]P-induced liver fibrosis in humans.

Various in vivo and in vitro experiments in human cell lines and rodents had shown that B[a]P induces different downstream processes through the activation of AhR (Tsai et al. 2015; Lou et al. 2022; Jin et al. 2022; Almendarez-Reyna et al. 2023). Subsequently, B[a]P exposure has been observed to induce oxidative stress in cells through increased interleukin 6 (IL-6) production as a result of activated NF-κB signaling pathway (Malik et al. 2018; Jin et al. 2022; Zheng et al. 2022). The oxidative stress caused by B[a]P exposure has been studied as a cause for disruption of lysosomes, eventually leading to dysfunctional autophagy (Gorria et al. 2008; Li et al. 2023). Finally, it has been shown that B[a]P exposure can induce different fibrotic pathways, including dysfunctional autophagy, in human hepatic models (Yan et al. 2021; Hill and Wang 2022). In conclusion, by leveraging various published evidence, we were able to explore a potential toxicity pathway that links B[a]P-induced toxicity with liver fibrosis in humans.

#### Toxicity pathway linking B[a]P exposure to early life-stage mortality in aquatic organisms

B[a]P is found in large quantities in different aquatic environments due to various anthropogenic activities and waste discharges from both household and industries (Bukowska et al. 2022; Sathikumaran et al. 2022). B[a]P is a toxic pollutant, and drastically affects various aquatic organisms, including economically relevant fish (Nacci et al. 2002; Seemann et al. 2015; Sathikumaran et al. 2022). Here, we observed that the AOP titled ‘Aryl hydrocarbon receptor activation leading to early life stage mortality via sox9 repression induced impeded craniofacial development’ (AOP:455), with biological applicability for developmental effects in aquatic species, has been identified as a B[a]P–AOP (Table S11). Moreover, this B[a]P–AOP is part of the largest connected component, and is currently included in the OECD work plan (Table S3). Therefore, we relied on this AOP to understand the rationale behind B[a]P-induced ecotoxicological effects in aquatic organisms.

Independent in vivo experiments in zebrafish and clam have shown that B[a]P exposure alters gene expression patterns through activation of AhR and subsequent dimerization of AhR and ARNT in affected tissues (Bugiak and Weber 2009; Wang et al. 2020). It has been shown that B[a]P exposure in zebrafish facilitates the recruitment of AhR-dependent long noncoding RNA (*slincR*) to *sox9b* 5′ UTR, eventually repressing its transcription (Garcia et al. 2018). *sox9b* is an important transcription factor involved in chondrocyte differentiation during zebrafish development (Dalcq et al. 2012). Subsequently it has been shown that B[a]P exposure induces alteration in expression patterns of genes involved in chondrogenesis, thereby leading to improper craniofacial skeleton development and eventually early life stage mortality in zebrafish (He et al. 2011; Seemann et al. 2015). In conclusion, by leveraging various published evidence, we were able to explore a potential toxicity pathway that links B[a]P-induced toxicity with early life stage mortality in aquatic species.

### Exploration of toxicity pathways in AOP network constructed from bisphenol A-relevant AOPs

Similar to the construction of B[a]P–AOP network, we constructed the AOP network for bisphenol A (BPA) toxicity. We identified 27 highly relevant AOPs associated with BPA-induced toxicities (Table 1), which we designated as BPA–AOPs. Among the 27 BPA–AOPs, we observed that 10 have ‘High’ cumulative WoE and 6 have ‘Moderate’ cumulative WoE (Table S14). In the undirected AOP network constructed from these 27 BPA–AOPs (Fig. S7), we observed five connected components (with two or more AOPs) and five isolated nodes, where the largest connected component (labeled C1) comprises 10 BPA–AOPs.

**Fig. 7:**
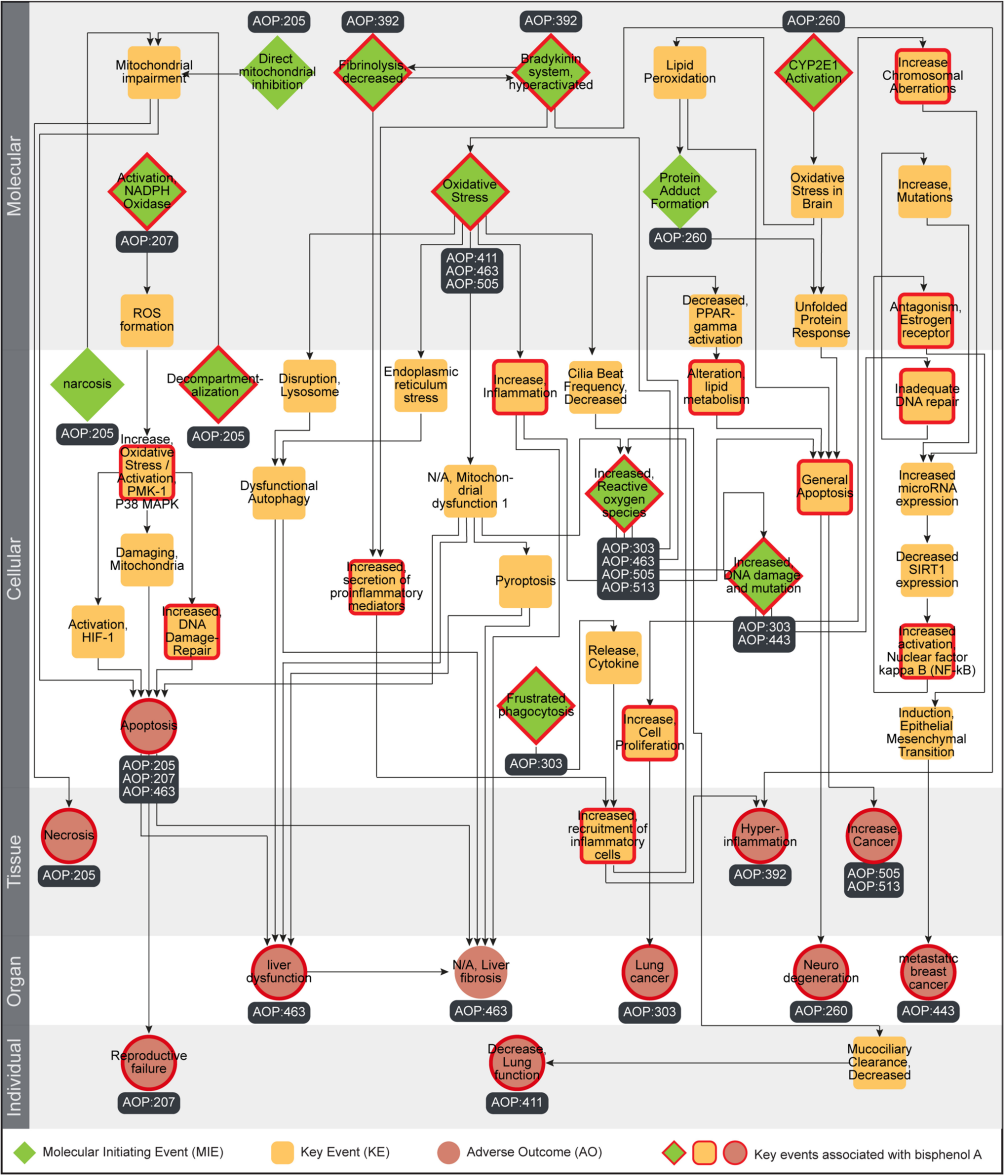
Directed network corresponding to the largest component in the undirected BPA–AOP network comprising 55 KEs and 72 KERs. Among the 55 KEs, 12 are categorized as MIEs (denoted as diamond), 11 are categorized as AOs (denoted as circle), and the remaining 32 are categorized as KEs (denoted as rounded square). The 31 KEs (including MIEs and AOs) associated with BPA are marked in ‘red’. In this figure, the 55 KEs are arranged vertically according to their level of biological organization (colour figure online)

We constructed and visualized a directed AOP network to explore the interactions among the BPA–AOPs present in the largest connected component C1 (Fig. 7). We observed that the directed network comprised 55 unique KEs (including 12 MIEs and 11 AOs) and 72 unique KERs (Fig. 7; Table S15). Among the 55 KEs, 31 KEs were associated with BPA-induced toxicity through our systematic data integrative approach, of which 9 are MIEs and 10 are AOs (Fig. 7).

Then, we computed several node-centric measures for the constructed BPA–AOP directed network. We observed that the AO ‘Apoptosis’ (KE:1262) and ‘N/A, Liver fibrosis’ (KE:344) have the highest in-degree of 5, while the MIE ‘Oxidative Stress’ (KE:1392) has the highest out-degree of 5 (Table S15). The MIE ‘Increased, Reactive oxygen species’ (KE:1115) has the highest betweenness centrality value, denoting that several toxicity pathways are passing through it in this network (Fig. S8; Table S15). The MIEs ‘Bradykinin system, hyperactivated’ (KE:1867), ‘Fibrinolysis, decreased’ (KE:1866), and ‘Frustrated phagocytosis’ (KE:1668) have the highest eccentricity, denoting that they are the most remotely placed KEs in this network (Fig. S9; Table S15). Finally, following a similar approach taken in the B[a]P–AOP directed network analysis, we compiled auxiliary evidence from published literature for all these 55 KEs (Table S16), and performed two case studies to explore both the human-relevant and ecotoxicology-relevant BPA-induced toxicity pathways from this directed BPA–AOP network.

#### Toxicity pathway linking BPA exposure to neurodegeneration in humans

Neurodegeneration refers to the progressive loss of structure or function of neurons, including their death (Hague et al. 2005; Przedborski 2008). This process is a characteristic feature of various neurological diseases, such as Alzheimer's disease, Parkinson's disease, and Huntington's disease (Hague et al. 2005; Przedborski 2008). Here, we observed BPA–AOP titled ‘CYP2E1 activation and formation of protein adducts leading to neurodegeneration’ (AOP:260) having a cumulative WoE of ‘High’ (Table S14) and taxonomical relevance to humans (Table S14). Therefore, we relied on this AOP to understand the rationale behind BPA-induced neurodegenerative effects.

BPA has been shown to induce the cytochrome P450 enzyme CYP2E1 in rat liver and kidney cells in both in vitro and in vivo studies (Hanioka et al. 2000; Shi et al. 2021). Although there is no direct evidence of BPA inducing CYP2E1 in the brain, BPA is known to cross the blood–brain barrier and cause adverse effects (Costa and Cairrao 2024). In an earlier study, Valencia-Olvera et al. (2014) identified that xenobiotic stress triggers CYP2E1 expression in cerebellar granule neurons, leading to oxidative stress through the generation of reactive oxygen species (ROS). This oxidative stress can activate apoptotic pathways through unfolded protein response in these neurons, which ultimately results in neurodegeneration (DeGracia et al. 2002; Coimbra-Costa et al. 2017). In conclusion, by leveraging various published evidence, we were able to explore a potential toxicity pathway that links BPA-induced toxicity with neurodegeneration in humans.

#### Toxicity pathway linking BPA exposure to reproductive failure in aquatic species

To understand the ecotoxicological effects of BPA exposure, we investigated the toxicity pathway outlined in the BPA–AOP titled 'NADPH oxidase and P38 MAPK activation leading to reproductive failure in Caenorhabditis elegans' (AOP:207). Although this AOP is documented as taxonomically applicable to *C. elegans* (Table S15), we identified auxiliary evidence supporting this toxicity pathway in aquatic species. Various in vitro assays have demonstrated that BPA exposure can lead to oxidative stress due to increased ROS production through the activation of NADPH oxidase (NOX) (Bosch-Panadero et al. 2018; Xia et al. 2022). In addition, BPA exposure has been observed to cause a significant increase in p38 MAPK levels in zebrafish ovarian cells (Biswas et al. 2020). This increase triggers ovarian inflammation and activates apoptotic pathways in zebrafish oocytes, ultimately leading to reduced reproductive success (Biswas et al. 2020). In conclusion, by leveraging various published evidence, we were able to explore a potential toxicity pathway that links BPA-induced toxicity with reproductive failure in aquatic organisms.

### Exploration of toxicity pathways in AOP network constructed from bis(2-ethylhexyl) phthalate-relevant AOPs

Similar to the construction of B[a]P–AOP network, we constructed the AOP network for bis(2-ethylhexyl) phthalate (CAS:117-81-7, commonly known as diethylhexyl phthalate or DEHP) toxicity. We identified 19 highly relevant AOPs associated with DEHP-induced toxicities (Table 1), which we designated as DEHP–AOPs. Among the 19 DEHP–AOPs, we observed that 6 have ‘High’ cumulative WoE and 6 have ‘Moderate’ cumulative WoE (Table S17). In the undirected AOP network constructed from these 19 DEHP–AOPs (Fig. S10), we observed four connected components (with two or more AOPs) and four isolated nodes, where the largest component (labeled C1) comprises 5 DEHP–AOPs.

We observed that the 5 DEHP–AOPs in the largest connected component C1 (AOP:263, AOP:264, AOP:265, AOP:267, and AOP:268) have ‘Decrease, Coupling of oxidative phosphorylation’ (KE:1446) as MIE and ‘Decrease, Growth’ (KE:1521) as AO. Notably, these 5 AOPs were developed by a single research group to address ecotoxicological effects on growth and development of organisms, and thus can be considered as potential alternate strategies to understand the same toxicity pathway (Sahoo et al. 2024). The connected components C2 and C3 are the next largest components, each containing 4 DEHP–AOPs. Therefore, we constructed and visualized the directed AOP networks to explore the interactions among DEHP–AOPs present in both C2 and C3 connected components.

We observed that the directed AOP network of the connected component C2 comprised 20 unique KEs (including 4 MIEs and 5 AOs) and 29 unique KERs (Fig. S11; Table S18). Among the 20 KEs, 11 KEs were associated with DEHP-induced toxicity through our systematic data integrative approach, of which 2 are MIEs and 4 are AOs (Fig. S11). Then we computed several node-centric network measures for this directed network. We observed that the AO ‘N/A, Liver fibrosis’ (KE:344) has the highest in-degree of 5, while the KEs ‘N/A, Mitochondrial dysfunction 1’ (KE:177) and ‘Oxidative Stress’ (KE:1392) have the highest out-degree of 4 (Table S18). The KE ‘Oxidative Stress’ (KE:1392) has the highest betweenness centrality value, denoting that several toxicity pathways are passing through it in this network (Fig. S12; Table S18). The ‘N/A, Mitochondrial dysfunction 1’ (KE:177) has the highest eccentricity, denoting that it is the most remotely placed KE in this network (Fig. S13; Table S18). Finally, following a similar approach taken in the B[a]P–AOP directed network analysis, we compiled auxiliary evidence from published literature for all these 20 KEs (Table S19), and performed a case study to explore human-relevant DEHP-induced toxicity pathway from this directed DEHP–AOP network.

We further constructed and visualized the directed AOP network of the connected component C3 and observed that it comprised 20 unique KEs (including 2 MIEs and 5 AOs) and 23 unique KERs (Fig. S14; Table S20). Among the 20 KEs, 14 KEs were associated with DEHP-induced toxicity through our systematic data integrative approach, of which 2 are MIEs and 3 are AOs (Fig. S14). Then we computed several node-centric network measures for this directed network. We observed that the KE ‘Increase, hepatocellular adenomas and carcinomas’ (KE:719) has the highest in-degree of 4, while the MIE ‘Activation, PPARα’ (KE:227) has the highest out-degree of 6 (Table S20). The KEs ‘Reduction, Cholesterol transport in mitochondria’ (KE:447) and ‘Reduction, Testosterone synthesis in Leydig cells’ (KE:413) have the highest betweenness centrality value, denoting that several toxicity pathways are passing through them in this network (Fig. S15; Table S20). The MIE ‘Activation, PPARα’ (KE:227) has the highest eccentricity, denoting that it is the most remotely placed KE in this network (Fig. S16; Table S20). Finally, following a similar approach taken in the B[a]P–AOP directed network analysis, we compiled auxiliary evidence from published literature for all these 20 KEs (Table S21), and performed a case study to explore ecotoxicology-relevant DEHP-induced toxicity pathway from this directed DEHP–AOP network.

#### Toxicity pathway linking DEHP exposure to liver dysfunction in humans

DEHP exposure has been associated with hepatotoxicity in humans, but the underlying toxicity pathway is not well understood (Zhao et al. 2019; Li et al. 2021a). Thus, we focused on the AOP titled ‘The AOP framework on silica nanoparticles induced hepatoxicity’ (AOP:463) to understand the toxicity pathway associated with DEHP-induced hepatotoxicity. Previous studies have shown that DEHP induces oxidative stress in rat hepatocytes due to excessive production of reactive oxygen species (ROS) (Zhao et al. 2019; Amara et al. 2020; Li et al. 2021a). This increased oxidative stress in hepatocytes induces pathways underlying mitochondrial dysfunction and inflammation (Zhao et al. 2019; Li et al. 2021a). Such pathways damage the hepatocytes, eventually leading to liver dysfunction in the DEHP-exposed rats (Zhao et al. 2019; Li et al. 2021a). In conclusion, by leveraging various published evidence, we were able to explore a potential toxicity pathway that links DEHP-induced toxicity with liver dysfunction in humans.

#### Toxicity pathway linking DEHP exposure to decreased population growth in aquatic species

To understand the ecotoxicological effects of DEHP exposure, we investigated the toxicity pathway outlined in the DEHP–AOP titled ‘PPARalpha Agonism Leading to Decreased Viable Offspring via Decreased 11-Ketotestosterone’ (AOP:323) having a cumulative WoE of ‘High’ (Table S17) and taxonomical relevance to teleost fish (Table S17). In DEHP-exposed fish, DEHP is metabolized into mono-ethylhexyl phthalate (MEHP), which preferentially binds to and activates the PPARalpha receptor (Maloney and Waxman 1999; Lapinskas et al. 2005; Sant et al. 2021). The activation of PPARalpha promotes lipid catabolism in the heart and cholesterol uptake in the liver, leading to an overall reduction in cholesterol levels (Maloney and Waxman 1999). Golshan et al. (2015) observed a significant reduction in 11-ketotestosterone (11-KT) levels in DEHP-treated goldfish. Similar reductions in 11-KT levels have been shown to impair spermatogenesis in male zebrafish, significantly affecting the viability of offspring and consequently hindering population growth (Zhang et al. 2020). In conclusion, by leveraging various published evidence, we were able to explore a potential toxicity pathway that links DEHP-induced toxicity with decreased population growth in aquatic species.

## Conclusion

Plastic additives are chemicals that are potentially released into the environment from various plastic products. The lack of information on their presence in various plastic products pose a challenge in evaluating their risks, thereby hindering proper regulatory measures. In this study, we constructed a stressor-centric AOP network of plastic additives to aid in their regulatory decision making. First, we relied on the UN report titled ‘Chemicals in Plastics—A Technical Report’ and identified 6470 plastic additives based on the reported chemical functions. Next, we systematically integrated heterogenous toxicogenomics and biological endpoints data from five exposome-relevant resources namely, ToxCast, CTD, DEDuCT, NeurotoxKb, and AOP–Wiki and identified 688 KEs within AOP–Wiki to be associated with 1314 plastic additives. Furthermore, we systematically curated 328 high confidence AOPs from AOP–Wiki and linked them to plastic additives based on overlapping KE associations. Here, we identified 322 high confidence AOPs to be associated with 1287 plastic additives while AOP–Wiki only documented 37 of the 1287 plastic additives to be associated with 27 of the 322 high confidence AOPs. Next, we constructed the stressor–AOP network for plastic additives (designated as plastic additives–AOP network) with varying levels of associations, where the plastic additives are categorized into ten priority use sectors and the AOPs are linked with 27 disease classes. We visualized the plastic additives–AOP network for each of the 1287 plastic additives and made them available in a dedicated website: https://cb.imsc.res.in/saopadditives/. Finally, we showed the utility of the constructed plastic additives–AOP network by identifying highly relevant AOPs (with Level 5 relevance and coverage score threshold of 0.4) associated with plastic additives. In particular, we identified highly relevant AOPs associated with benzo[a]pyrene (B[a]P), bisphenol A (BPA), and bis(2-ethylhexyl) phthalate (DEHP) and relied on published experimental evidence to explore human- and ecotoxicology-relevant toxicity pathways.

However, the functional annotations of chemicals as plastic additives provided by the UNEP report may be inaccurate (Wiesinger et al. 2021). For example, B[a]P is annotated as a plasticizer, cross-linker, lubricant, and filler in the UNEP report, but it has been reported as a byproduct or a contaminant resulting from the use of other plastic additives during plastic production (Lassen et al. 2011; Alassali et al. 2020). Similarly, other chemicals may have been misidentified due to inaccuracies in the functional annotations provided by the UNEP report. Moreover, due to limited information on their presence in various use sectors, we were able to identify only 4309 of the 6470 plastic additives across ten priority use sectors. Furthermore, due to the paucity of plastic additive exposure studies, we were able to associate only 1287 of the 6470 plastic additives to AOPs within AOP–Wiki. We observed that 197 of the 322 high confidence AOPs (associated with the 1287 plastic additives) capture toxicity pathways leading to human relevant adverse effects. Moreover, the toxicogenomics approach followed in this study relied majorly on mammalian-centric biological data, thereby limiting its scope to explore various ecotoxicological events.

Nonetheless, we have constructed the first and most comprehensive stressor–AOP network for plastic additives that has enabled the exploration of different toxicity mechanisms underlying plastic additives-specific adverse outcomes. Furthermore, we observed that many of these plastic additives are produced in high volumes globally and are documented to cause endocrine disruptions. Notably, we observed that these additives can accumulate in various human tissues as xenobiotics, suggesting that prolonged exposure to plastic additives can lead to highly deleterious effects in different organ systems (Sorci and Loiseau 2022; Maddela et al. 2023). Importantly, the constructed plastic additives–AOP network was useful in the identification of highly relevant AOPs for plastic additives which highlighted plastic additives-induced emergent toxicity pathways. The scope of the present work can be expanded by leveraging larger and well curated data sets of chemicals found in plastics (Wagner et al. 2024), additional information on gene targets, chemicals, diseases, pathways and species associated with the existing AOPs (Pittman et al. 2018), and non-human biological endpoints (Olker et al. 2022; Kramer et al. 2024) for ecologically relevant species. In sum, this is the first study that utilizes the AOP framework to explore the various adverse effects associated with plastic additives, assisting their risk assessment and contributing towards their regulatory decision-making.

## Supplementary Information

Below is the link to the electronic supplementary material.

Supplementary file1 (PDF 4095 KB)

Supplementary file2 (XLSX 7896 KB)

## Data Availability

The data associated with this study is contained in the article or in the supplementary information files or in the associated website: https://cb.imsc.res.in/saopadditives/.

## References

[CR1] Aguayo-Orozco A, Audouze K, Siggaard T et al (2019) sAOP: linking chemical stressors to adverse outcomes pathway networks. Bioinformatics 35:5391–5392. 10.1093/bioinformatics/btz570 31329252 10.1093/bioinformatics/btz570 PMC9887475

[CR2] Alassali A, Calmano W, Gidarakos E, Kuchta K (2020) The degree and source of plastic recyclates contamination with polycyclic aromatic hydrocarbons. RSC Adv 10:44989–44996. 10.1039/D0RA08554E 35516248 10.1039/D0RA08554E PMC9058816

[CR3] Al-Malaika S, Axtell F, Rothon R, Gilbert M (2017) Chapter 7 - additives for plastics. In: Gilbert M (ed) Brydson’s plastics materials, 8th edn. Butterworth-Heinemann, pp 127–168

[CR4] Almendarez-Reyna CI, de la Trinidad Chacón CG, Ochoa-Martínez ÁC et al (2023) The aryl hydrocarbon receptor (AhR) activation mediates benzo(a)pyrene-induced overexpression of AQP3 and Notch1 in HaCaT cells. Environ Mol Mutagen 64:466–472. 10.1002/em.22580 37984337 10.1002/em.22580

[CR5] Amara I, Timoumi R, Annabi E et al (2020) Di (2-ethylhexyl) phthalate targets the thioredoxin system and the oxidative branch of the pentose phosphate pathway in liver of Balb/c mice. Environ Toxicol 35:78–86. 10.1002/tox.22844 31486570 10.1002/tox.22844

[CR6] Ankley GT, Bennett RS, Erickson RJ et al (2010) Adverse outcome pathways: a conceptual framework to support ecotoxicology research and risk assessment. Environ Toxicol Chem 29:730–741. 10.1002/etc.34 20821501 10.1002/etc.34

[CR7] Aurisano N, Huang L, Milài Canals L et al (2021a) Chemicals of concern in plastic toys. Environ Int 146:106194 33115697 10.1016/j.envint.2020.106194

[CR8] Aurisano N, Weber R, Fantke P (2021b) Enabling a circular economy for chemicals in plastics. Curr Opin Green Sustain Chem 31:100513. 10.1016/j.cogsc.2021.100513 10.1016/j.cogsc.2021.100513

[CR9] Baker N, Knudsen T, Williams A (2017) Abstract sifter: a comprehensive front-end system to PubMed. F1000Research 6:2164. 10.12688/f1000research.12865.1 10.12688/f1000research.12865.1 PMC5801564 29479422

[CR10] Barouki R, Samson M, Blanc EB et al (2023) The exposome and liver disease - how environmental factors affect liver health. J Hepatol 79:492–505. 10.1016/j.jhep.2023.02.034 36889360 10.1016/j.jhep.2023.02.034 PMC10448911

[CR11] BASF (2013) Additives for adhesives and sealants. https://www.basf.com/global/documents/en/products-and-industries/architectural-coatings/20130501_Additives_for_Adhesives_and_Sealants_Catalogue_Europe.pdf.assetinline.pdf Accessed 15 Feb 2024

[CR12] Bataller R, Brenner DA (2005) Liver fibrosis. J Clin Investig 115:209–218. 10.1172/JCI24282 15690074 10.1172/JCI24282 PMC546435

[CR13] Biswas S, Ghosh S, Samanta A et al (2020) Bisphenol A impairs reproductive fitness in zebrafish ovary: Potential involvement of oxidative/nitrosative stress, inflammatory and apoptotic mediators. Environ Pollut 267:115692. 10.1016/j.envpol.2020.115692 33254711 10.1016/j.envpol.2020.115692

[CR14] Bosch-Panadero E, Mas S, Civantos E et al (2018) Bisphenol A is an exogenous toxin that promotes mitochondrial injury and death in tubular cells. Environ Toxicol 33:325–332. 10.1002/tox.22519 29214717 10.1002/tox.22519

[CR15] Bugiak B, Weber LP (2009) Hepatic and vascular mRNA expression in adult zebrafish (Danio rerio) following exposure to benzo-a-pyrene and 2,3,7,8-tetrachlorodibenzo-p-dioxin. Aquat Toxicol 95:299–306. 10.1016/j.aquatox.2009.03.009 19403181 10.1016/j.aquatox.2009.03.009

[CR16] Bukowska B, Mokra K, Michałowicz J (2022) Benzo[a]pyrene—environmental occurrence, human exposure, and mechanisms of toxicity. Int J Mol Sci 23:6348. 10.3390/ijms23116348 35683027 10.3390/ijms23116348 PMC9181839

[CR17] Chai Z, Zhao C, Jin Y et al (2021) Generating adverse outcome pathway (AOP) of inorganic arsenic-induced adult male reproductive impairment via integration of phenotypic analysis in comparative toxicogenomics database (CTD) and AOP wiki. Toxicol Appl Pharmacol 411:115370. 10.1016/j.taap.2020.115370 33338516 10.1016/j.taap.2020.115370

[CR18] Cheung AC, Walker DI, Juran BD et al (2020) Studying the exposome to understand the environmental determinants of complex liver diseases. Hepatology 71:352–362. 10.1002/hep.31028 31701542 10.1002/hep.31028 PMC7329010

[CR19] Coimbra-Costa D, Alva N, Duran M et al (2017) Oxidative stress and apoptosis after acute respiratory hypoxia and reoxygenation in rat brain. Redox Biol 12:216–225. 10.1016/j.redox.2017.02.014 28259102 10.1016/j.redox.2017.02.014 PMC5334548

[CR20] Coleman EA (2017) 21 - plastics additives. In: Kutz M (ed) Applied plastics engineering handbook, 2nd edn. William Andrew Publishing, Norwich, pp 489–500

[CR21] Costa HE, Cairrao E (2024) Effect of bisphenol A on the neurological system: a review update. Arch Toxicol 98:1–73. 10.1007/s00204-023-03614-0 37855918 10.1007/s00204-023-03614-0 PMC10761478

[CR22] da Costa JP, Avellan A, Mouneyrac C et al (2023) Plastic additives and microplastics as emerging contaminants: mechanisms and analytical assessment. TrAC Trends Anal Chem 158:116898. 10.1016/j.trac.2022.116898 10.1016/j.trac.2022.116898

[CR23] Dalcq J, Pasque V, Ghaye A et al (2012) RUNX3, EGR1 and SOX9B form a regulatory cascade required to modulate BMP-signaling during cranial cartilage development in zebrafish. PLoS ONE 7:e50140. 10.1371/journal.pone.0050140 23209659 10.1371/journal.pone.0050140 PMC3507947

[CR24] Davis AP, Wiegers TC, Grondin CJ et al (2020) Leveraging the comparative toxicogenomics database to fill in knowledge gaps for environmental health: a test case for air pollution-induced cardiovascular disease. Toxicol Sci 177:392–404. 10.1093/toxsci/kfaa113 32663284 10.1093/toxsci/kfaa113 PMC7548289

[CR25] Davis AP, Wiegers TC, Johnson RJ et al (2023) Comparative toxicogenomics database (CTD): update 2023. Nucleic Acids Res 51:D1257–D1262. 10.1093/nar/gkac833 36169237 10.1093/nar/gkac833 PMC9825590

[CR26] De Frond H, Rubinovitz R, Rochman CM (2021) μATR-FTIR spectral libraries of plastic particles (FLOPP and FLOPP-e) for the analysis of microplastics. Anal Chem 93:15878–15885. 10.1021/acs.analchem.1c02549 34813292 10.1021/acs.analchem.1c02549

[CR27] DeGracia DJ, Kumar R, Owen CR et al (2002) Molecular pathways of protein synthesis inhibition during brain reperfusion: implications for neuronal survival or death. J Cereb Blood Flow Metab 22:127–140. 10.1097/00004647-200202000-00001 11823711 10.1097/00004647-200202000-00001

[CR28] Dionisio KL, Phillips K, Price PS et al (2018) The Chemical and products database, a resource for exposure-relevant data on chemicals in consumer products. Sci Data 5:180125. 10.1038/sdata.2018.125 29989593 10.1038/sdata.2018.125 PMC6038847

[CR29] Dix DJ, Houck KA, Martin MT et al (2007) The ToxCast program for prioritizing toxicity testing of environmental chemicals. Toxicol Sci 95:5–12. 10.1093/toxsci/kfl103 16963515 10.1093/toxsci/kfl103

[CR30] EPA US (2023) ToxCast & Tox21 summary files from invitrodb_v4. Retrieved from https://www.epa.gov/chemical-research/toxicity-forecaster-toxcasttm-data. Accessed 31 Oct 2023. Data released September 2023

[CR31] Feshuk M, Kolaczkowski L, Dunham K et al (2023) The ToxCast pipeline: updates to curve-fitting approaches and database structure. Front Toxicol 5:1275980. 10.3389/ftox.2023.1275980 37808181 10.3389/ftox.2023.1275980 PMC10552852

[CR32] Fröhlich H, Speer N, Poustka A, Beißbarth T (2007) GOSim – an R-package for computation of information theoretic GO similarities between terms and gene products. BMC Bioinform 8:166. 10.1186/1471-2105-8-166 10.1186/1471-2105-8-166 PMC1892785 17519018

[CR33] Garcia GR, Shankar P, Dunham CL et al (2018) Signaling events downstream of AHR activation that contribute to toxic responses: the functional role of an AHR-dependent long noncoding RNA (*slincR*) using the zebrafish model. Environ Health Perspect 126:117002. 10.1289/EHP3281 30398377 10.1289/EHP3281 PMC6371766

[CR34] Geyer R, Jambeck JR, Law KL (2017) Production, use, and fate of all plastics ever made. Sci Adv 3:e1700782. 10.1126/sciadv.1700782 28776036 10.1126/sciadv.1700782 PMC5517107

[CR35] Golshan M, Hatef A, Socha M et al (2015) Di-(2-ethylhexyl)-phthalate disrupts pituitary and testicular hormonal functions to reduce sperm quality in mature goldfish. Aquat Toxicol 163:16–26. 10.1016/j.aquatox.2015.03.017 25827748 10.1016/j.aquatox.2015.03.017

[CR36] Gorria M, Tekpli X, Rissel M et al (2008) A new lactoferrin- and iron-dependent lysosomal death pathway is induced by benzo[a]pyrene in hepatic epithelial cells. Toxicol Appl Pharmacol 228:212–224. 10.1016/j.taap.2007.12.021 18255115 10.1016/j.taap.2007.12.021

[CR37] Groh KJ, Backhaus T, Carney-Almroth B et al (2019) Overview of known plastic packaging-associated chemicals and their hazards. Sci Total Environ 651:3253–3268. 10.1016/j.scitotenv.2018.10.015 30463173 10.1016/j.scitotenv.2018.10.015

[CR38] Groh KJ, Geueke B, Martin O et al (2021) Overview of intentionally used food contact chemicals and their hazards. Environ Int 150:106225. 10.1016/j.envint.2020.106225 33272655 10.1016/j.envint.2020.106225

[CR39] Hagberg AA, Schult DA, Swart PJ (2008) Exploring network structure, dynamics, and function using NetworkX. In: Varoquaux G, Vaught T, Millman J (eds) Proceedings of the 7th python in science conference (SciPy 2008). pp 11–15

[CR40] Hague SM, Klaffke S, Bandmann O (2005) Neurodegenerative disorders: Parkinson’s disease and Huntington’s disease. J Neurol Neurosurg Psychiatry 76:1058–1063. 10.1136/jnnp.2004.060186 16024878 10.1136/jnnp.2004.060186 PMC1739745

[CR41] Hahladakis JN, Velis CA, Weber R et al (2018) An overview of chemical additives present in plastics: migration, release, fate and environmental impact during their use, disposal and recycling. J Hazard Mater 344:179–199. 10.1016/j.jhazmat.2017.10.014 29035713 10.1016/j.jhazmat.2017.10.014

[CR42] Hanioka N, Jinno H, Tanaka-Kagawa T et al (2000) Interaction of bisphenol A with rat hepatic cytochrome P450 enzymes. Chemosphere 41:973–978. 10.1016/S0045-6535(99)00529-9 10879814 10.1016/S0045-6535(99)00529-9

[CR43] He C, Zuo Z, Shi X et al (2011) Effects of benzo(a)pyrene on the skeletal development of *Sebastiscus marmoratus* embryos and the molecular mechanism involved. Aquat Toxicol 101:335–341. 10.1016/j.aquatox.2010.11.008 21216343 10.1016/j.aquatox.2010.11.008

[CR44] Hecker M, Peijnenburg W, Lam PKS, Brinkmann M (2023) Emerging issues in aquatic toxicology—plastic pollution. Aquat Toxicol 264:106729. 10.1016/j.aquatox.2023.106729 37872039 10.1016/j.aquatox.2023.106729

[CR45] Hermabessiere L, Dehaut A, Paul-Pont I et al (2017) Occurrence and effects of plastic additives on marine environments and organisms: a review. Chemosphere 182:781–793. 10.1016/j.chemosphere.2017.05.096 28545000 10.1016/j.chemosphere.2017.05.096

[CR46] Herrera A, Acosta-Dacal A, Pérez Luzardo O et al (2022) Bioaccumulation of additives and chemical contaminants from environmental microplastics in European seabass (*Dicentrarchus labrax*). Sci Total Environ 822:153396. 10.1016/j.scitotenv.2022.153396 35092768 10.1016/j.scitotenv.2022.153396

[CR47] Hill C, Wang Y (2022) Autophagy in pulmonary fibrosis: friend or foe? Genes Dis 9:1594–1607. 10.1016/j.gendis.2021.09.008 36119644 10.1016/j.gendis.2021.09.008 PMC7613590

[CR48] Jaylet T, Coustillet T, Jornod F et al (2023) AOP-helpFinder 2.0: Integration of an event-event searches module. Environ Int 177:108017. 10.1016/j.envint.2023.108017 37295163 10.1016/j.envint.2023.108017

[CR49] Jeong J, Choi J (2020) Development of AOP relevant to microplastics based on toxicity mechanisms of chemical additives using ToxCast™ and deep learning models combined approach. Environ Int 137:105557. 10.1016/j.envint.2020.105557 32078872 10.1016/j.envint.2020.105557

[CR50] Jeong J, Kim D, Choi J (2023) Integrative data mining approach: case study with adverse outcome pathway network leading to pulmonary fibrosis. Chem Res Toxicol 36:838–847. 10.1021/acs.chemrestox.2c00325 37093963 10.1021/acs.chemrestox.2c00325

[CR51] Jin Y, Qi G, Shou Y et al (2022) High throughput data-based, toxicity pathway-oriented development of a quantitative adverse outcome pathway network linking AHR activation to lung damages. J Hazard Mater 425:128041. 10.1016/j.jhazmat.2021.128041 34906874 10.1016/j.jhazmat.2021.128041

[CR52] Jornod F, Jaylet T, Blaha L et al (2022) AOP-helpFinder webserver: a tool for comprehensive analysis of the literature to support adverse outcome pathways development. Bioinformatics 38:1173–1175. 10.1093/bioinformatics/btab750 34718414 10.1093/bioinformatics/btab750 PMC8796376

[CR53] Judson R, Houck K, Martin M et al (2016) Analysis of the effects of cell stress and cytotoxicity on in vitro assay activity across a diverse chemical and assay Space. Toxicol Sci 152:323–339. 10.1093/toxsci/kfw092 27208079 10.1093/toxsci/kfw092 PMC6280881

[CR54] Karthikeyan BS, Ravichandran J, Mohanraj K et al (2019) A curated knowledgebase on endocrine disrupting chemicals and their biological systems-level perturbations. Sci Total Environ 692:281–296. 10.1016/j.scitotenv.2019.07.225 31349169 10.1016/j.scitotenv.2019.07.225

[CR55] Karthikeyan BS, Ravichandran J, Aparna SR, Samal A (2021) DEDuCT 2.0: an updated knowledgebase and an exploration of the current regulations and guidelines from the perspective of endocrine disrupting chemicals. Chemosphere 267:128898. 10.1016/j.chemosphere.2020.128898 33190914 10.1016/j.chemosphere.2020.128898

[CR56] Knapen D, Angrish MM, Fortin MC et al (2018) Adverse outcome pathway networks I: development and applications. Environ Toxicol Chem 37:1723–1733. 10.1002/etc.4125 29488651 10.1002/etc.4125 PMC6004608

[CR57] Koleske JV, Springate R, Brezinski D (2011) Additives handbook. Paint & Coatings Industry 6:1–64

[CR58] Kramer L, Schulze T, Klüver N et al (2024) Curated mode-of-action data and effect concentrations for chemicals relevant for the aquatic environment. Sci Data 11:60. 10.1038/s41597-023-02904-7 38200014 10.1038/s41597-023-02904-7 PMC10781676

[CR59] Lapinskas PJ, Brown S, Leesnitzer LM et al (2005) Role of PPARα in mediating the effects of phthalates and metabolites in the liver. Toxicology 207:149–163. 10.1016/j.tox.2004.09.008 15590130 10.1016/j.tox.2004.09.008

[CR60] Lassen P, Hoffmann L, Thomsen M (2011) PAHs in toys and childcare products. Environmental Protection Agency, Danish Ministry of the Environment, Denmark

[CR61] Leist M, Ghallab A, Graepel R et al (2017) Adverse outcome pathways: opportunities, limitations and open questions. Arch Toxicol 91:3477–3505. 10.1007/s00204-017-2045-3 29051992 10.1007/s00204-017-2045-3

[CR62] Lewis KA, Tzilivakis J, Warner DJ, Green A (2016) An international database for pesticide risk assessments and management. Hum Ecol Risk Assess Int J 22:1050–1064. 10.1080/10807039.2015.1133242 10.1080/10807039.2015.1133242

[CR63] Li G, Zhao C-Y, Wu Q et al (2021a) Integrated metabolomics and transcriptomics reveal di(2-ethylhexyl) phthalate-induced mitochondrial dysfunction and glucose metabolism disorder through oxidative stress in rat liver. Ecotoxicol Environ Saf 228:112988. 10.1016/j.ecoenv.2021.112988 34808505 10.1016/j.ecoenv.2021.112988

[CR64] Li P, Wang X, Su M et al (2021b) Characteristics of plastic pollution in the environment: a review. Bull Environ Contam Toxicol 107:577–584. 10.1007/s00128-020-02820-1 32166334 10.1007/s00128-020-02820-1

[CR65] Li J, Bai J, Si X et al (2023) Benzo[a]pyrene induces epithelial tight junction disruption and apoptosis via inhibiting the initiation of autophagy in intestinal porcine epithelial cells. Chem Biol Interact 374:110386. 10.1016/j.cbi.2023.110386 36754226 10.1016/j.cbi.2023.110386

[CR66] Lou W, Zhang M, Chen Q et al (2022) Molecular mechanism of benzo [a] pyrene regulating lipid metabolism via aryl hydrocarbon receptor. Lipids Health Dis 21:13. 10.1186/s12944-022-01627-9 35057794 10.1186/s12944-022-01627-9 PMC8772151

[CR67] Maddela NR, Kakarla D, Venkateswarlu K, Megharaj M (2023) Additives of plastics: entry into the environment and potential risks to human and ecological health. J Environ Manag 348:119364. 10.1016/j.jenvman.2023.119364 10.1016/j.jenvman.2023.119364 37866190

[CR68] Maes T, Preston-Whyte F, Lavelle S et al (2023) A recipe for plastic: expert insights on plastic additives in the marine environment. Mar Pollut Bull 196:115633. 10.1016/j.marpolbul.2023.115633 37864860 10.1016/j.marpolbul.2023.115633

[CR69] Malik D, David RM, Gooderham NJ (2018) Mechanistic evidence that benzo[a]pyrene promotes an inflammatory microenvironment that drives the metastatic potential of human mammary cells. Arch Toxicol 92:3223–3239. 10.1007/s00204-018-2291-z 30155724 10.1007/s00204-018-2291-z PMC6132703

[CR70] Maloney EK, Waxman DJ (1999) trans-activation of PPARα and PPARγ by structurally diverse environmental chemicals. Toxicol Appl Pharmacol 161:209–218. 10.1006/taap.1999.8809 10581215 10.1006/taap.1999.8809

[CR71] Meeker JD, Sathyanarayana S, Swan SH (2009) Phthalates and other additives in plastics: human exposure and associated health outcomes. Philos Transa R Soc b: Biol Sci 364:2097–2113. 10.1098/rstb.2008.0268 10.1098/rstb.2008.0268 PMC2873014 19528058

[CR72] Menger F, Boström G, Jonsson O et al (2021) Identification of pesticide transformation products in surface water using suspect screening combined with national monitoring data. Environ Sci Technol 55:10343–10353. 10.1021/acs.est.1c00466 34291901 10.1021/acs.est.1c00466 PMC8383268

[CR73] Nacci DE, Kohan M, Pelletier M, George E (2002) Effects of benzo[a]pyrene exposure on a fish population resistant to the toxic effects of dioxin-like compounds. Aquat Toxicol 57:203–215. 10.1016/S0166-445X(01)00196-5 11932001 10.1016/S0166-445X(01)00196-5

[CR74] National Research Council (2007) Toxicity testing in the 21st century: a vision and a strategy. The National Academies Press, Washington, DC. 10.17226/11970

[CR75] Neveu V, Nicolas G, Salek RM et al (2020) Exposome-explorer 2.0: an update incorporating candidate dietary biomarkers and dietary associations with cancer risk. Nucleic Acids Res 48:D908–D912. 10.1093/nar/gkz1009 31724701 10.1093/nar/gkz1009 PMC7145555

[CR76] OECD (2016) Users’ handbook supplement to the guidance document for developing and assessing adverse outcome pathways, OECD series on adverse outcome pathways No. 1. OECD Publishing, Paris. 10.1787/5jlv1m9d1g32-en

[CR77] OECD (2017) Revised guidance document on developing and assessing adverse outcome pathways, series on testing and assessment No. 184. OECD Publishing, Paris

[CR78] Oehlmann J, Oetken M, Schulte-Oehlmann U (2008) A critical evaluation of the environmental risk assessment for plasticizers in the freshwater environment in Europe, with special emphasis on bisphenol A and endocrine disruption. Environ Res 108:140–149. 10.1016/j.envres.2008.07.016 18949832 10.1016/j.envres.2008.07.016

[CR79] Olker JH, Elonen CM, Pilli A et al (2022) The ECOTOXicology knowledgebase: a curated database of ecologically relevant toxicity tests to support environmental research and risk assessment. Environ Toxicol Chem 41:1520–1539. 10.1002/etc.5324 35262228 10.1002/etc.5324 PMC9408435

[CR80] Parthasarathy A, Tyler AC, Hoffman MJ et al (2019) Is plastic pollution in aquatic and terrestrial environments a driver for the transmission of pathogens and the evolution of antibiotic resistance? Environ Sci Technol 53:1744–1745. 10.1021/acs.est.8b07287 30702278 10.1021/acs.est.8b07287

[CR81] Pittman ME, Edwards SW, Ives C, Mortensen HM (2018) AOP-DB: a database resource for the exploration of adverse outcome pathways through integrated association networks. Toxicol Appl Pharmacol 343:71–83. 10.1016/j.taap.2018.02.006 29454060 10.1016/j.taap.2018.02.006 PMC6104822

[CR82] Pogrmic-Majkic K, Samardzija Nenadov D, Tesic B et al (2022) Mapping DEHP to the adverse outcome pathway network for human female reproductive toxicity. Arch Toxicol 96:2799–2813. 10.1007/s00204-022-03333-y 35790550 10.1007/s00204-022-03333-y PMC9352620

[CR83] Pollesch NL, Villeneuve DL, O’Brien JM (2019) Extracting and benchmarking emerging adverse outcome pathway knowledge. Toxicol Sci 168:349–364. 10.1093/toxsci/kfz006 30715536 10.1093/toxsci/kfz006 PMC10545168

[CR84] Pritchard G (ed) (2012) Plastics additives an A-Z reference. Springer Science, Business Media Dordrecht, Cham

[CR85] Przedborski S (2008) Neurodegeneration. In: Gendelman HE, Ikezu T (eds) Neuroimmune pharmacology. Springer, Boston, pp 229–237

[CR86] Qian S, Ji H, Wu X et al (2018) Detection and quantification analysis of chemical migrants in plastic food contact products. PLoS ONE 13:e0208467. 10.1371/journal.pone.0208467 30517180 10.1371/journal.pone.0208467 PMC6281260

[CR87] Ravichandran J, Karthikeyan BS, Aparna SR, Samal A (2021a) Network biology approach to human tissue-specific chemical exposome. J Steroid Biochem Mol Biol 214:105998. 10.1016/j.jsbmb.2021.105998 34534667 10.1016/j.jsbmb.2021.105998

[CR88] Ravichandran J, Karthikeyan BS, Singla P et al (2021b) NeurotoxKb 1.0: compilation, curation and exploration of a knowledgebase of environmental neurotoxicants specific to mammals. Chemosphere 278:130387. 10.1016/j.chemosphere.2021.130387 33838427 10.1016/j.chemosphere.2021.130387

[CR89] Ravichandran J, Karthikeyan BS, Jost J, Samal A (2022a) An atlas of fragrance chemicals in children’s products. Sci Total Environ 818:151682. 10.1016/j.scitotenv.2021.151682 34793786 10.1016/j.scitotenv.2021.151682

[CR90] Ravichandran J, Karthikeyan BS, Samal A (2022b) Investigation of a derived adverse outcome pathway (AOP) network for endocrine-mediated perturbations. Sci Total Environ 826:154112. 10.1016/j.scitotenv.2022.154112 35219661 10.1016/j.scitotenv.2022.154112

[CR91] Rugard M, Coumoul X, Carvaillo J-C et al (2020) Deciphering adverse outcome pathway network linked to bisphenol f using text mining and systems toxicology approaches. Toxicol Sci 173:32–40. 10.1093/toxsci/kfz214 31596483 10.1093/toxsci/kfz214 PMC6944215

[CR92] Saarimäki LA, Fratello M, Pavel A et al (2023) A curated gene and biological system annotation of adverse outcome pathways related to human health. Sci Data 10:409. 10.1038/s41597-023-02321-w 37355733 10.1038/s41597-023-02321-w PMC10290716

[CR93] Sahoo AK, Chivukula N, Ramesh K et al (2024) An integrative data-centric approach to derivation and characterization of an adverse outcome pathway network for cadmium-induced toxicity. Sci Total Environ 920:170968. 10.1016/j.scitotenv.2024.170968 38367714 10.1016/j.scitotenv.2024.170968

[CR94] Sant KE, Moreau HM, Williams LM et al (2021) Embryonic exposures to mono-2-ethylhexyl phthalate induce larval steatosis in zebrafish independent of Nrf2a signaling. J Dev Orig Health Dis 12:132–140. 10.1017/S2040174420000057 32063256 10.1017/S2040174420000057 PMC7429360

[CR95] Sathikumaran R, Madhuvandhi J, Priya K et al (2022) Evaluation of benzo[a]pyrene-induced toxicity in the estuarine thornfish *Therapon jarbua*. Toxicol Rep 9:720–727. 10.1016/j.toxrep.2022.03.051 36518429 10.1016/j.toxrep.2022.03.051 PMC9742828

[CR96] Schriml LM, Munro JB, Schor M et al (2022) The human disease ontology 2022 update. Nucleic Acids Res 50:D1255–D1261. 10.1093/nar/gkab1063 34755882 10.1093/nar/gkab1063 PMC8728220

[CR97] Seemann F, Peterson DR, Witten PE et al (2015) Insight into the transgenerational effect of benzo[a]pyrene on bone formation in a teleost fish (*Oryzias latipes*). Comp Biochem Physiol c: Toxicol Pharmacol 178:60–67. 10.1016/j.cbpc.2015.10.001 26456900 10.1016/j.cbpc.2015.10.001

[CR98] Sendra M, Pereiro P, Figueras A, Novoa B (2021) An integrative toxicogenomic analysis of plastic additives. J Hazard Mater 409:124975. 10.1016/j.jhazmat.2020.124975 33388451 10.1016/j.jhazmat.2020.124975

[CR99] Shannon P, Markiel A, Ozier O et al (2003) Cytoscape: a software environment for integrated models of biomolecular interaction networks. Genome Res 13:2498–2504. 10.1101/gr.1239303 14597658 10.1101/gr.1239303 PMC403769

[CR100] Shi R, Liu Z, Liu T (2021) The antagonistic effect of bisphenol A and nonylphenol on liver and kidney injury in rats. Immunopharmacol Immunotoxicol 43:527–535. 10.1080/08923973.2021.1950179 34282716 10.1080/08923973.2021.1950179

[CR101] Sorci G, Loiseau C (2022) Should we worry about the accumulation of microplastics in human organs? EBioMedicine 82:104191. 10.1016/j.ebiom.2022.104191 35907367 10.1016/j.ebiom.2022.104191 PMC9335379

[CR102] Stevens S, McPartland M, Bartosova Z et al (2024) Plastic food packaging from five countries contains endocrine- and metabolism-disrupting chemicals. Environ Sci Technol 58:4859–4871. 10.1021/acs.est.3c08250 38441001 10.1021/acs.est.3c08250 PMC10956434

[CR103] Takes FW, Kosters WA (2011) Determining the diameter of small world networks. In: Proceedings of the 20th ACM international conference on information and knowledge management. association for computing machinery, New York, NY, USA, pp 1191–1196

[CR104] Thompson RC, Moore CJ, vom Saal FS, Swan SH (2009) Plastics, the environment and human health: current consensus and future trends. Philos Trans Royal Soc b: Biol Sci 364:2153–2166. 10.1098/rstb.2009.0053 10.1098/rstb.2009.0053 PMC2873021 19528062

[CR105] Tsai C-H, Li C-H, Liao P-L et al (2015) NcoA2-dependent inhibition of HIF-1α activation is regulated via AhR. Toxicol Sci 148:517–530. 10.1093/toxsci/kfv199 26350169 10.1093/toxsci/kfv199

[CR106] UNEP (2023) Chemicals in plastics - a technical report. https://www.unep.org/resources/report/chemicals-plastics-technical-report. Accessed 5 Feb 2024

[CR107] Valencia-Olvera AC, Morán J, Camacho-Carranza R et al (2014) CYP2E1 induction leads to oxidative stress and cytotoxicity in glutathione-depleted cerebellar granule neurons. Toxicol in Vitro 28:1206–1214. 10.1016/j.tiv.2014.05.014 24929095 10.1016/j.tiv.2014.05.014

[CR108] Villeneuve DL, Crump D, Garcia-Reyero N et al (2014a) Adverse outcome pathway (AOP) development I: strategies and principles. Toxicol Sci 142:312–320. 10.1093/toxsci/kfu199 25466378 10.1093/toxsci/kfu199 PMC4318923

[CR109] Villeneuve DL, Crump D, Garcia-Reyero N et al (2014b) Adverse outcome pathway development II: best practices. Toxicol Sci 142:321–330. 10.1093/toxsci/kfu200 25466379 10.1093/toxsci/kfu200 PMC4318924

[CR110] Villeneuve DL, Angrish MM, Fortin MC et al (2018) Adverse outcome pathway networks II: network analytics. Environ Toxicol Chem 37:1734–1748. 10.1002/etc.4124 29492998 10.1002/etc.4124 PMC6010347

[CR111] Vinken M, Knapen D, Vergauwen L et al (2017) Adverse outcome pathways: a concise introduction for toxicologists. Arch Toxicol 91:3697–3707. 10.1007/s00204-017-2020-z 28660287 10.1007/s00204-017-2020-z PMC5805086

[CR112] Wagner M, Monclús L, Arp HPH, Groh KJ, Løseth ME, Muncke J, Wang Z, Wolf R, Zimmermann L (2024) State of the science on plastic chemicals - identifying and addressing chemicals and polymers of concern. 10.5281/zenodo.10701706

[CR113] Wang H, Pan L, Zhang X et al (2020) The molecular mechanism of AhR-ARNT-XREs signaling pathway in the detoxification response induced by polycyclic aromatic hydrocarbons (PAHs) in clam *Ruditapes* philippinarum. Environ Res 183:109165. 10.1016/j.envres.2020.109165 32032812 10.1016/j.envres.2020.109165

[CR114] Wiesinger H, Wang Z, Hellweg S (2021) Deep dive into plastic monomers, additives, and processing aids. Environ Sci Technol 55:9339–9351. 10.1021/acs.est.1c00976 34154322 10.1021/acs.est.1c00976

[CR115] Williams A (2021) The chemical and products database (CPDat) MySQL data file. 10.23645/EPACOMPTOX.5352997

[CR116] Williams AJ, Grulke CM, Edwards J et al (2017) The CompTox chemistry dashboard: a community data resource for environmental chemistry. J Cheminform 9:61. 10.1186/s13321-017-0247-6 29185060 10.1186/s13321-017-0247-6 PMC5705535

[CR117] Williams AJ, Lambert JC, Thayer K, Dorne J-LCM (2021) Sourcing data on chemical properties and hazard data from the US-EPA CompTox chemicals dashboard: a practical guide for human risk assessment. Environ Int 154:106566. 10.1016/j.envint.2021.106566 33934018 10.1016/j.envint.2021.106566 PMC9667884

[CR118] Xia T, Guo J, Zhang B et al (2022) Bisphenol A promotes the progression of colon cancer through dual-targeting of NADPH oxidase and mitochondrial electron-transport chain to produce ROS and activating HIF-1α/VEGF/PI3K/AKT axis. Front Endocrinol 13:933051. 10.3389/fendo.2022.933051 10.3389/fendo.2022.933051 PMC9289207 35860704

[CR119] Yan L, Messner CJ, Zhang X, Suter-Dick L (2021) Assessment of fibrotic pathways induced by environmental chemicals using 3D-human liver microtissue model. Environ Res 194:110679. 10.1016/j.envres.2020.110679 33387535 10.1016/j.envres.2020.110679

[CR120] Yang W, Cui H, Chai Z et al (2022) Benzo[a]pyrene inhibits testosterone biosynthesis via NDUFA10-mediated mitochondrial compromise in mouse Leydig cells: integrating experimental and in silico toxicological approaches. Ecotoxicol Environ Saf 244:114075. 10.1016/j.ecoenv.2022.114075 36108438 10.1016/j.ecoenv.2022.114075

[CR121] Zhang Q, Ye D, Wang H et al (2020) Zebrafish cyp11c1 knockout reveals the roles of 11-ketotestosterone and cortisol in sexual development and reproduction. Endocrinology 161:bqaa048. 10.1210/endocr/bqaa048 32222764 10.1210/endocr/bqaa048

[CR122] Zhao Z, Ji K, Shen X et al (2019) Di(2-ethylhexyl) phthalate promotes hepatic fibrosis by regulation of oxidative stress and inflammation responses in rats. Environ Toxicol Pharmacol 68:109–119. 10.1016/j.etap.2019.03.008 30884453 10.1016/j.etap.2019.03.008

[CR123] Zheng Z, Park JK, Kwon OW et al (2022) The Risk of gastrointestinal cancer on daily intake of low-dose BaP in C57BL/6 for 60 days. Korean Acad Med Sci 37:e235. 10.3346/jkms.2022.37.e235 10.3346/jkms.2022.37.e235 PMC9344036 35916047

