# Supplementary material for: Leveraging integrative toxicogenomic approach towards development of stressor-centric adverse outcome pathway networks for plastic additives
Source: Arch Toxicol. 2024 Aug 3;98(10):3299–321. doi: 10.1007/s00204-024-03825-z (PMC11402864; doi:10.1007/s00204-024-03825-z)
Supplement: Supplementary file 1 — Supplementary file1 (PDF 4095 KB) [file 204_2024_3825_MOESM1_ESM.pdf]

<u>Supplementary Figures S1-S16</u>

<u>for</u>

Leveraging integrative toxicogenomic approach towards development of stressor-centric adverse outcome pathway networks for plastic additives

Ajaya Kumar Sahoo[a,b,1], Nikhil Chivukula[a,b,1], Shreyes Rajan Madgaonkar[a,b], Kundhanathan Ramesh[a], Shambanagouda Rudragouda Marigoudar[c], Krishna Venkatarama Sharma[c], Areejit Samal[a,b,*]

[a] *The Institute of Mathematical Sciences (IMSc), Chennai, India*

[b] *Homi Bhabha National Institute (HBNI), Mumbai, India*

[c] *National Centre for Coastal Research, Ministry of Earth Sciences, Government of India, Pallikaranai, Chennai, India*

[1]A.K.S. and N.C. contributed equally to this work and should be considered as Joint-First authors

*Corresponding author: asamal@imsc.res.in (A. Samal)

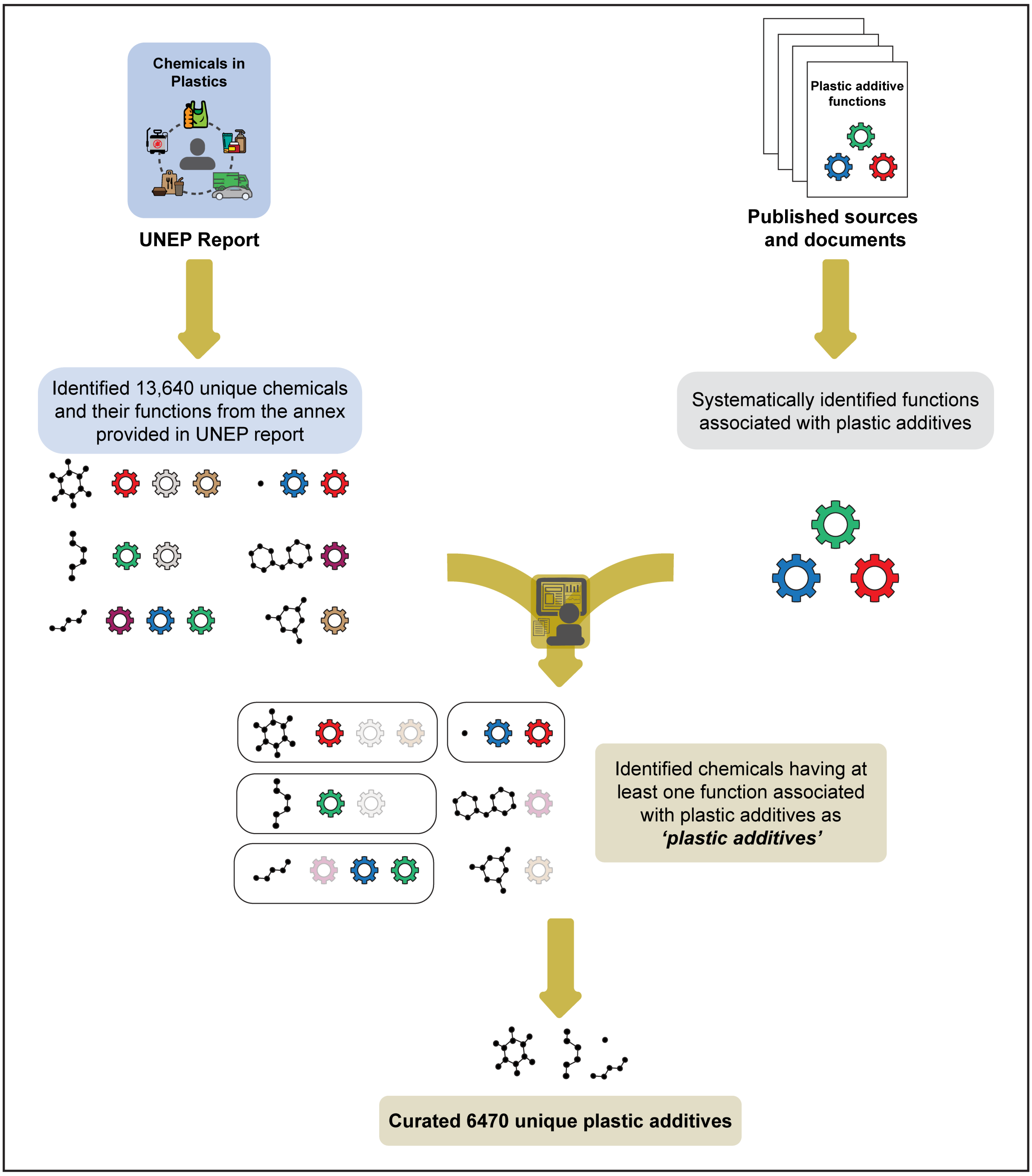

Fig. S1 Workflow to identify 6470 unique plastic additives from chemicals documented in the UNEP report titled 'Chemicals in Plastics – A Technical Report' (https://www.unep.org/resources/report/chemicals-plastics-technical-report).

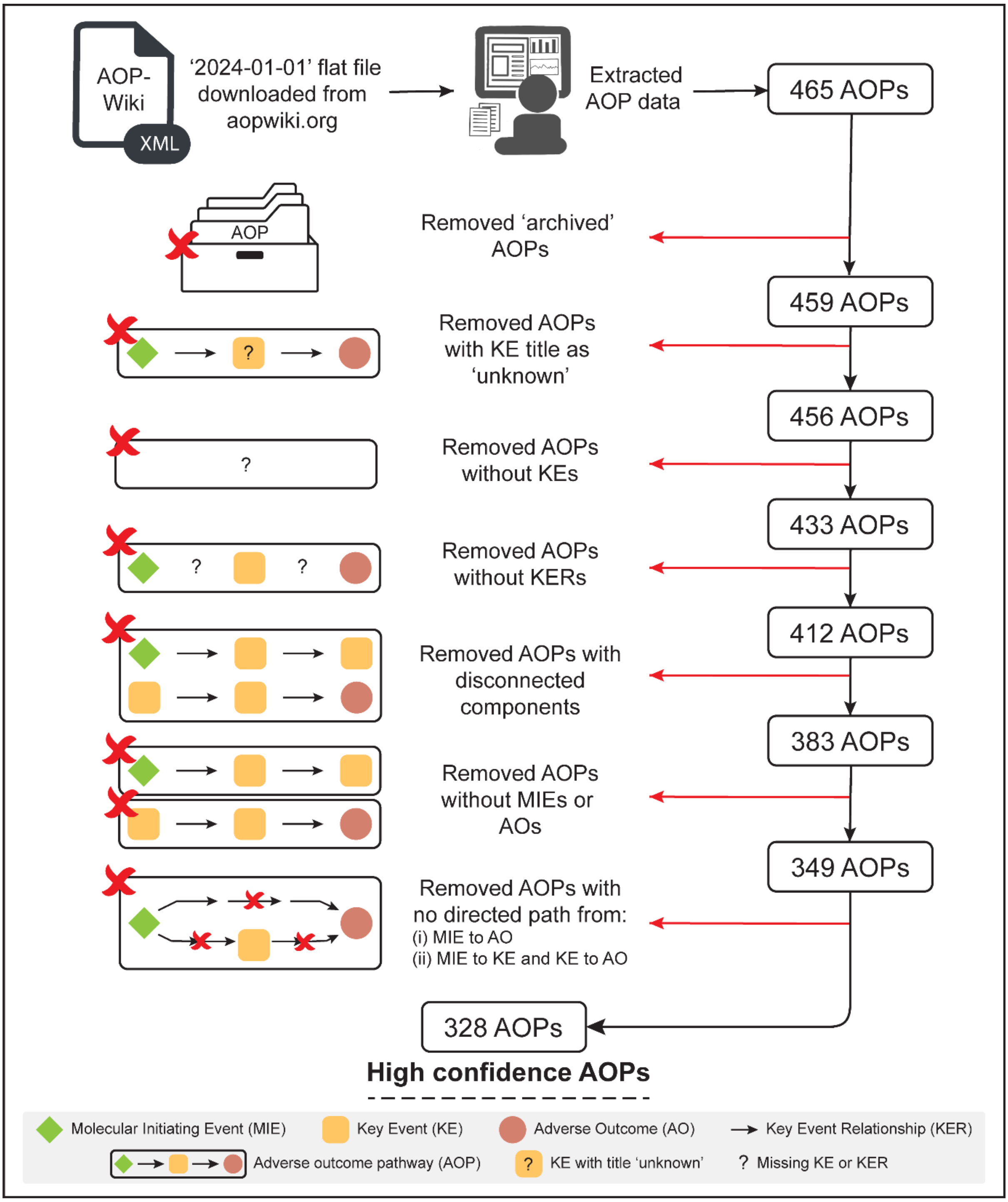

Fig. S2 Workflow to filter high confidence adverse outcome pathways (AOPs) from AOP-Wiki by employing computation and manual curation in conjunction. (Adapted from Sahoo *et al.*, 2024)

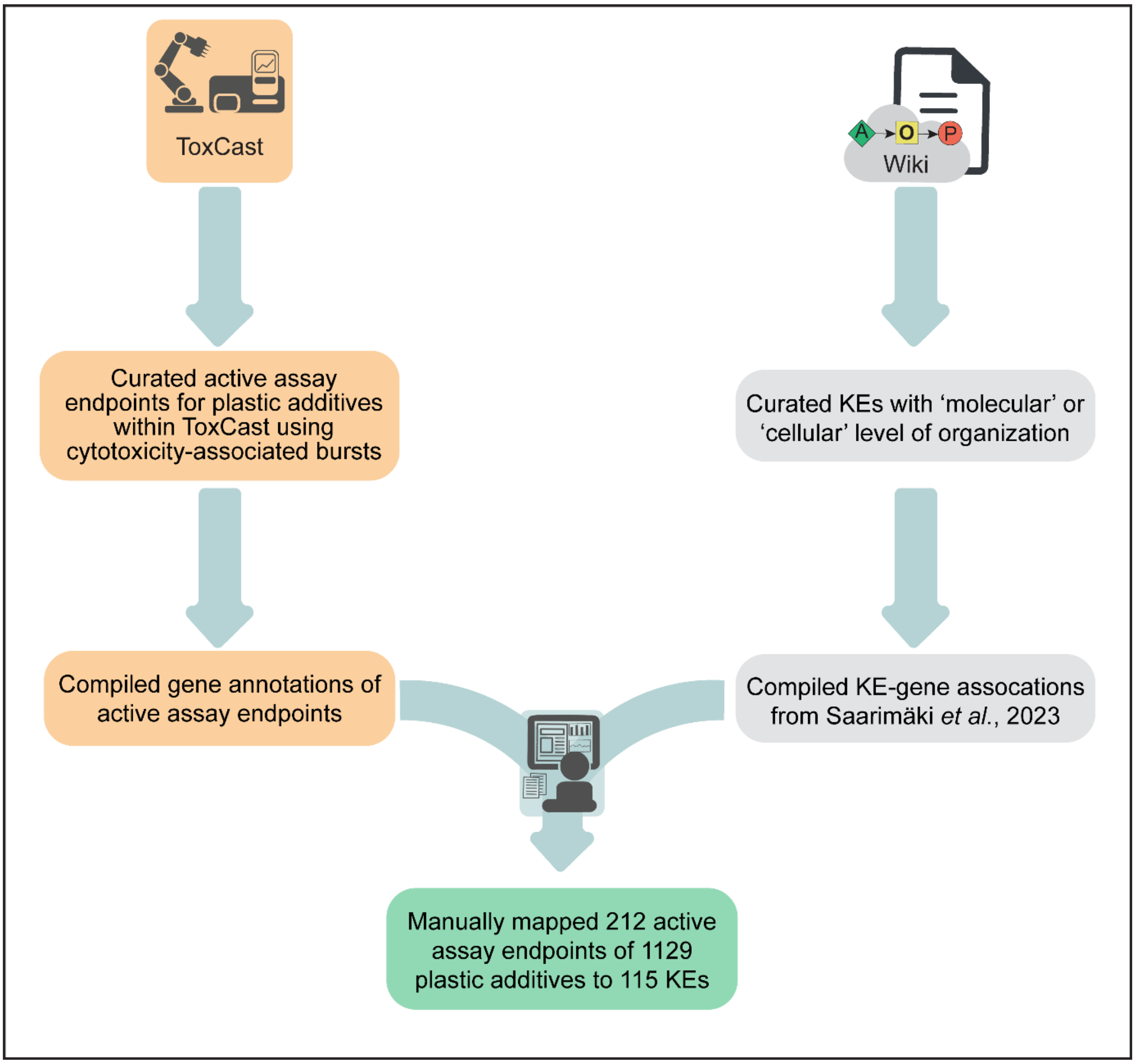

Fig. S3 Workflow to identify KEs from AOP-Wiki which are mapped to the active assay endpoints of plastic additives within ToxCast.

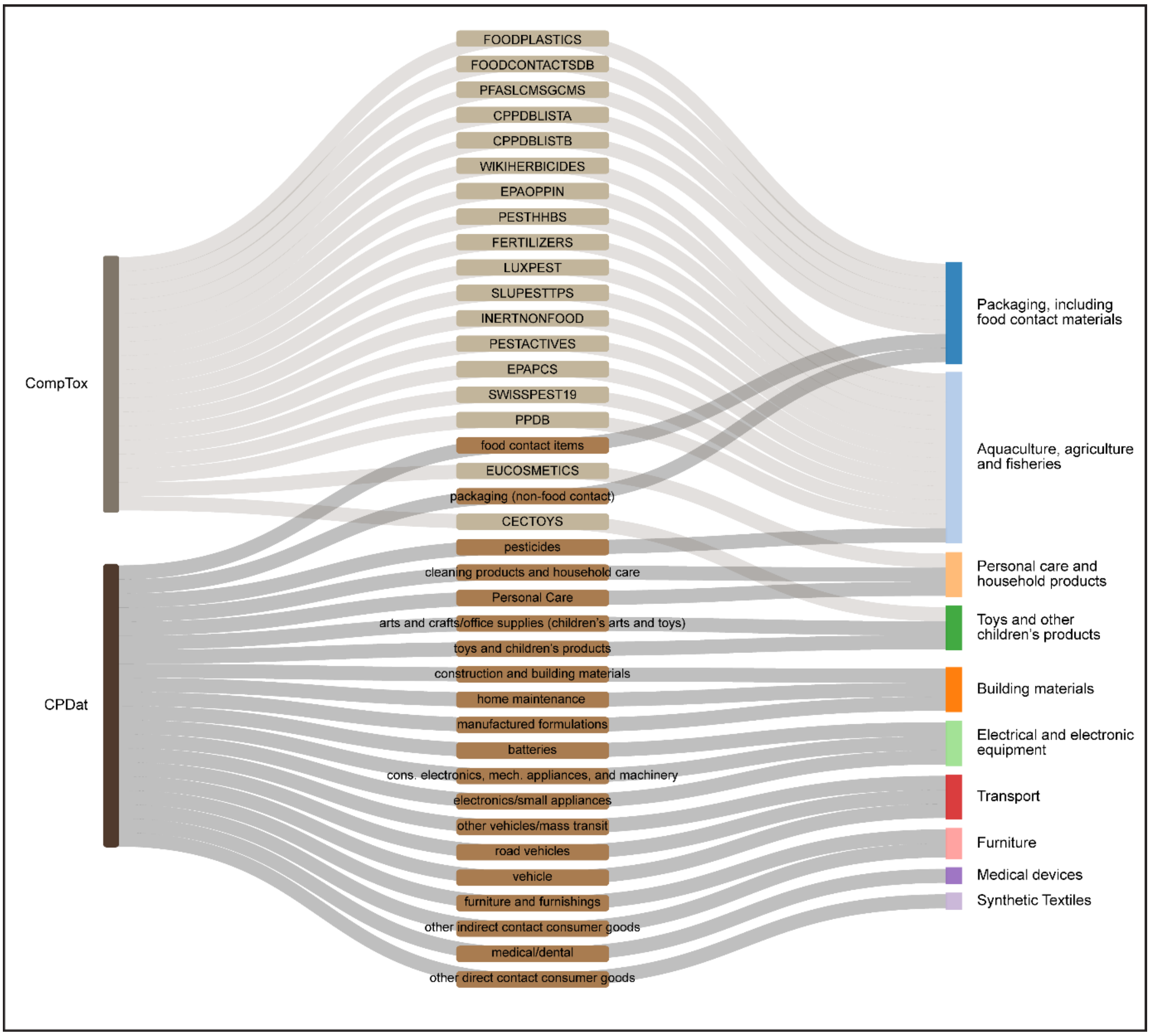

Fig. S4 Mapping of chemical or category lists from CompTox Chemicals Dashboard and Chemical and Products Database (CPDat) with the 10 priority use sectors of plastic additives.

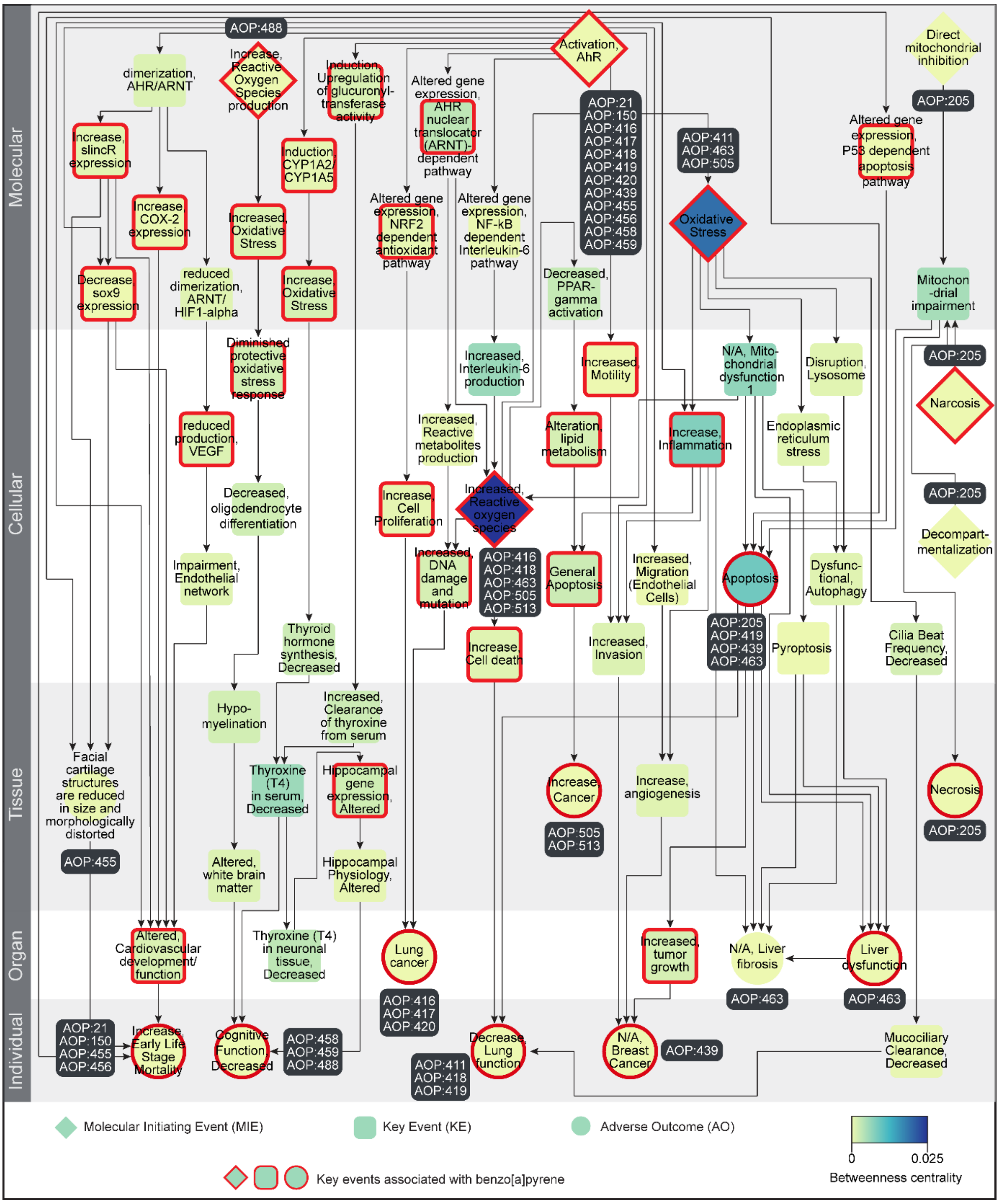

Fig. S5 Directed network corresponding to the largest connected component (C1) in the B[a]P-AOP network, where the KEs (including MIEs and AOs) are colored based on their betweenness centrality values. The 36 KEs (including MIEs and AOs) associated with B[a]P are marked in 'red'. In this figure, the 66 KEs are arranged vertically according to their level of biological organization.

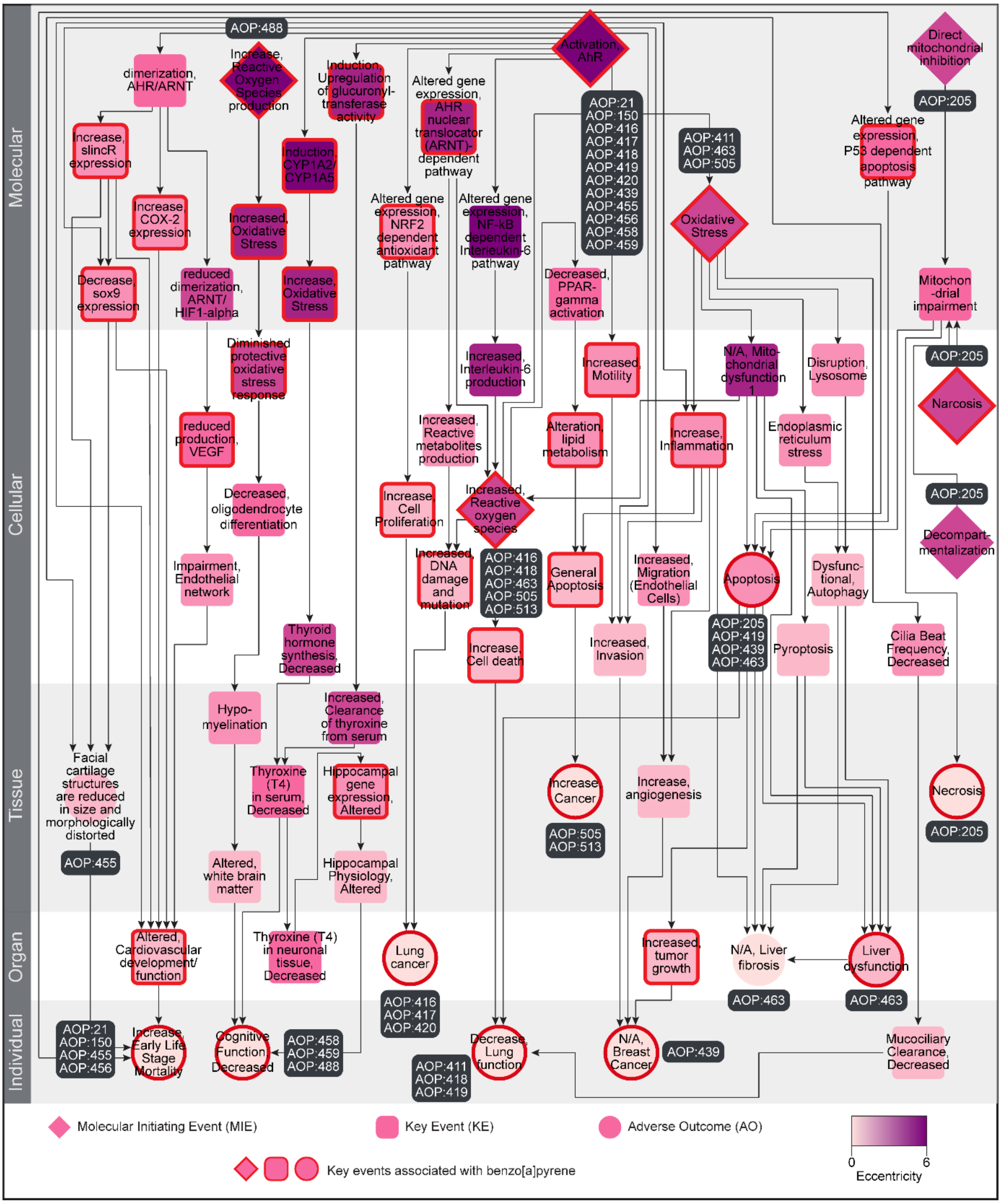

Fig. S6 Directed network corresponding to the largest connected component (C1) in the B[a]P-AOP network, where the KEs (including MIEs and AOs) are colored based on their eccentricity values. The 36 KEs (including MIEs and AOs) associated with B[a]P are marked in 'red'. In this figure, the 66 KEs are arranged vertically according to their level of biological organization.

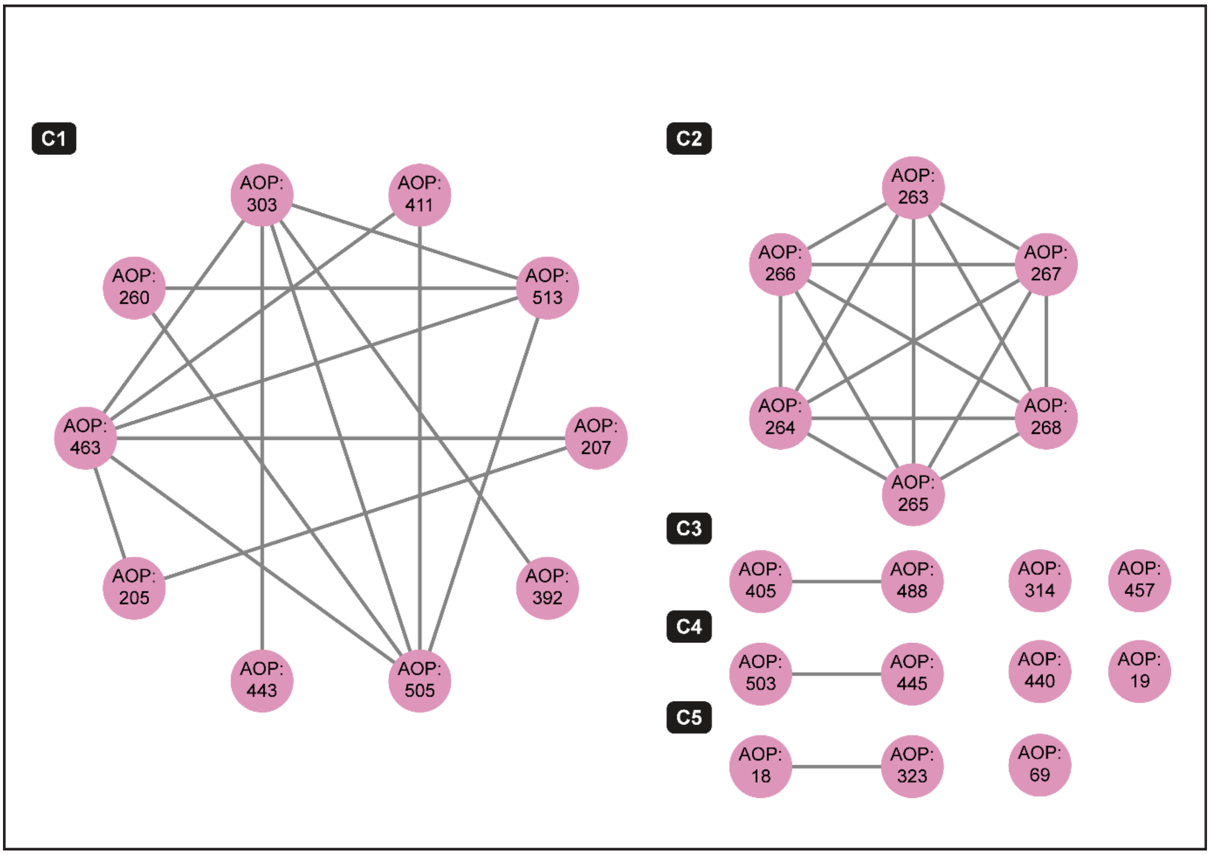

Fig. S7 Undirected network of bisphenol A (BPA)-AOPs. Each node corresponds to BPA-AOP and an edge between two nodes denotes that the two AOPs share at least one KE. This undirected network has 5 connected components (with two or more nodes) which are labeled as C1, C2, C3, C4 and C5, and 5 isolated nodes.

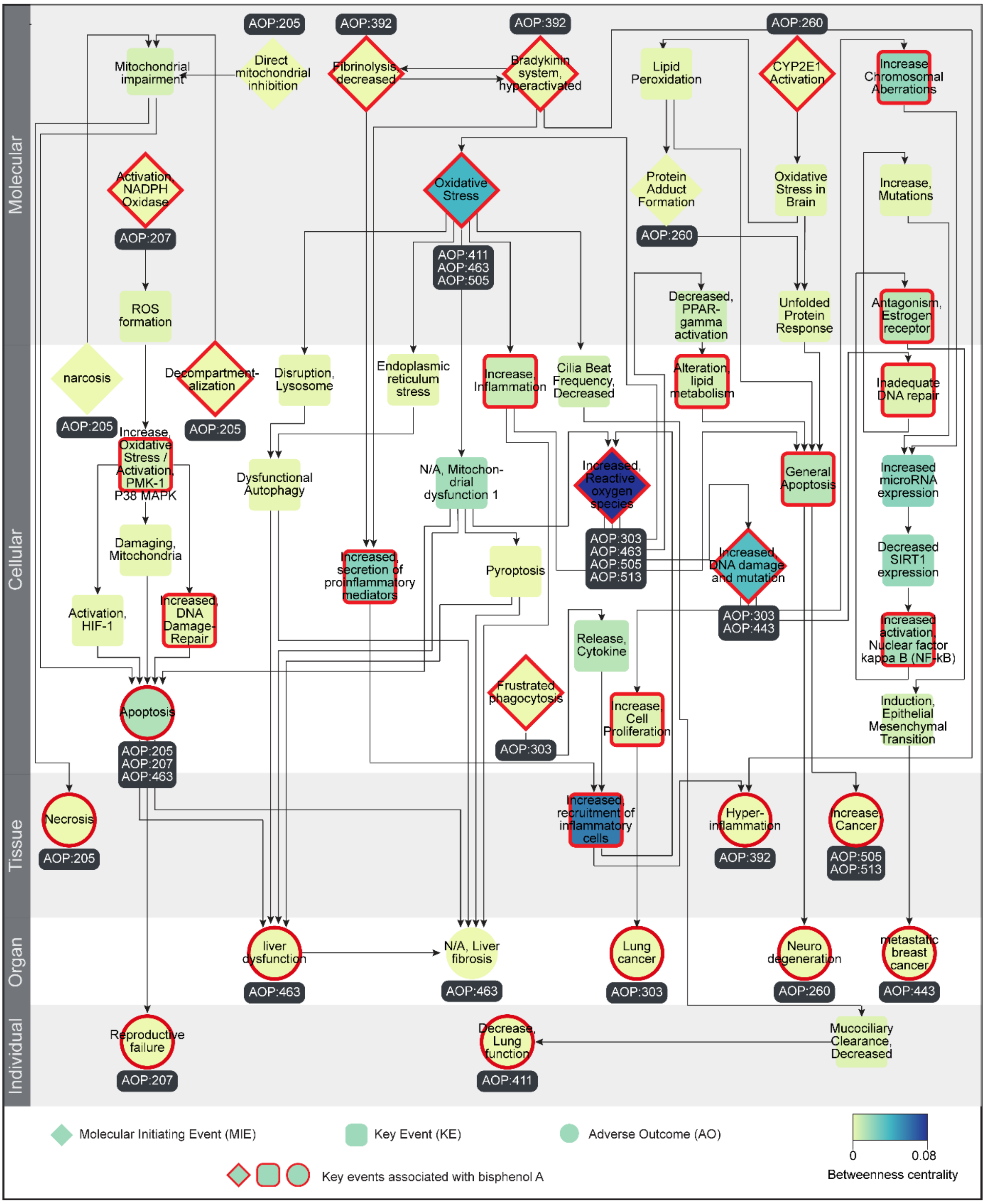

Fig. S8 Directed network corresponding to the largest connected component (C1) in the BPA-AOP network, where the KEs (including MIEs and AOs) are colored based on their betweenness centrality values. The 31 KEs (including MIEs and AOs) associated with BPA are marked in 'red'. In this figure, the 55 KEs are arranged vertically according to their level of biological organization.

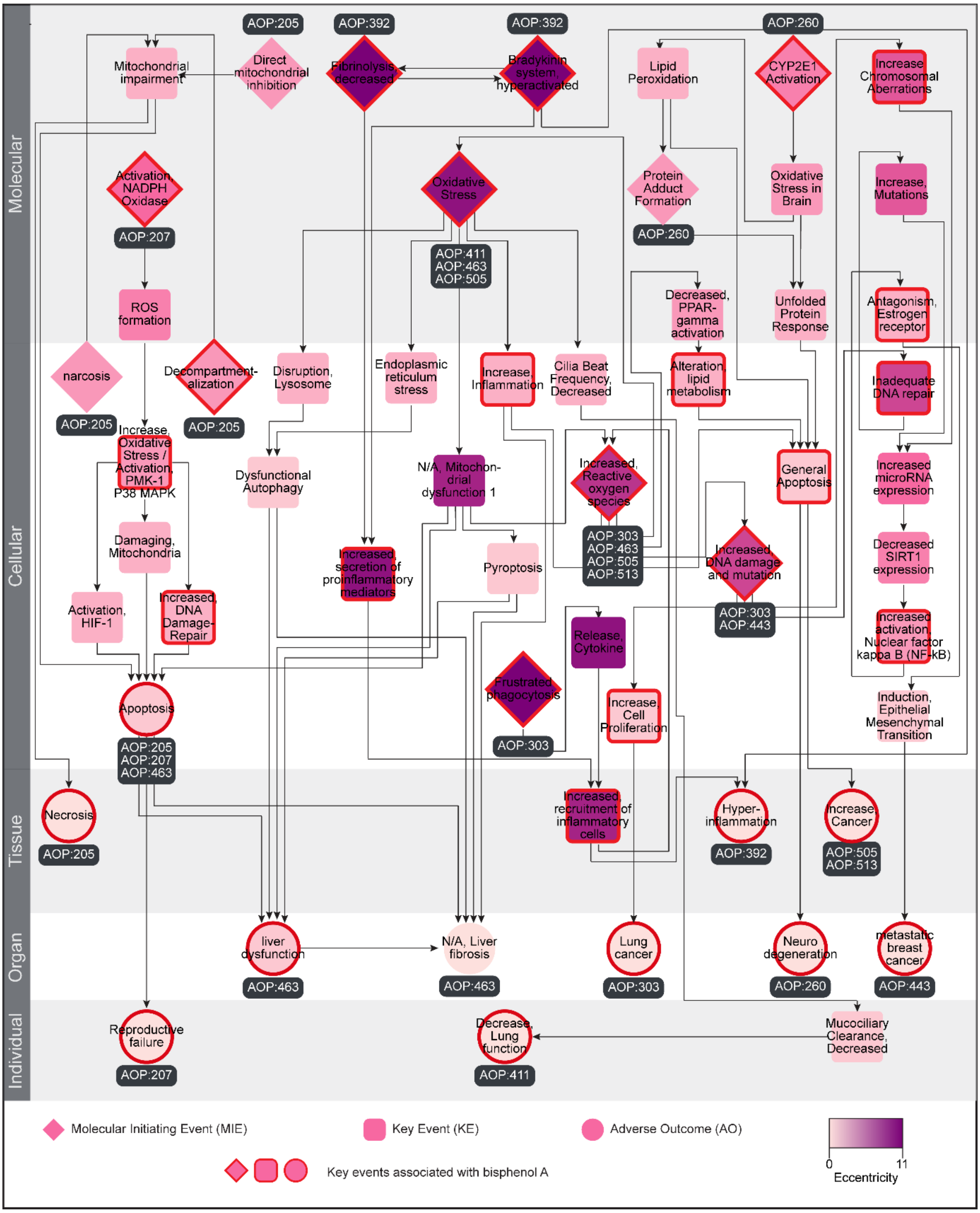

Fig. S9 Directed network corresponding to the largest connected component (C1) in the BPA-AOP network, where the KEs (including MIEs and AOs) are colored based on their eccentricity values. The 31 KEs (including MIEs and AOs) associated with BPA are marked in 'red'. In this figure, the 55 KEs are arranged vertically according to their level of biological organization.

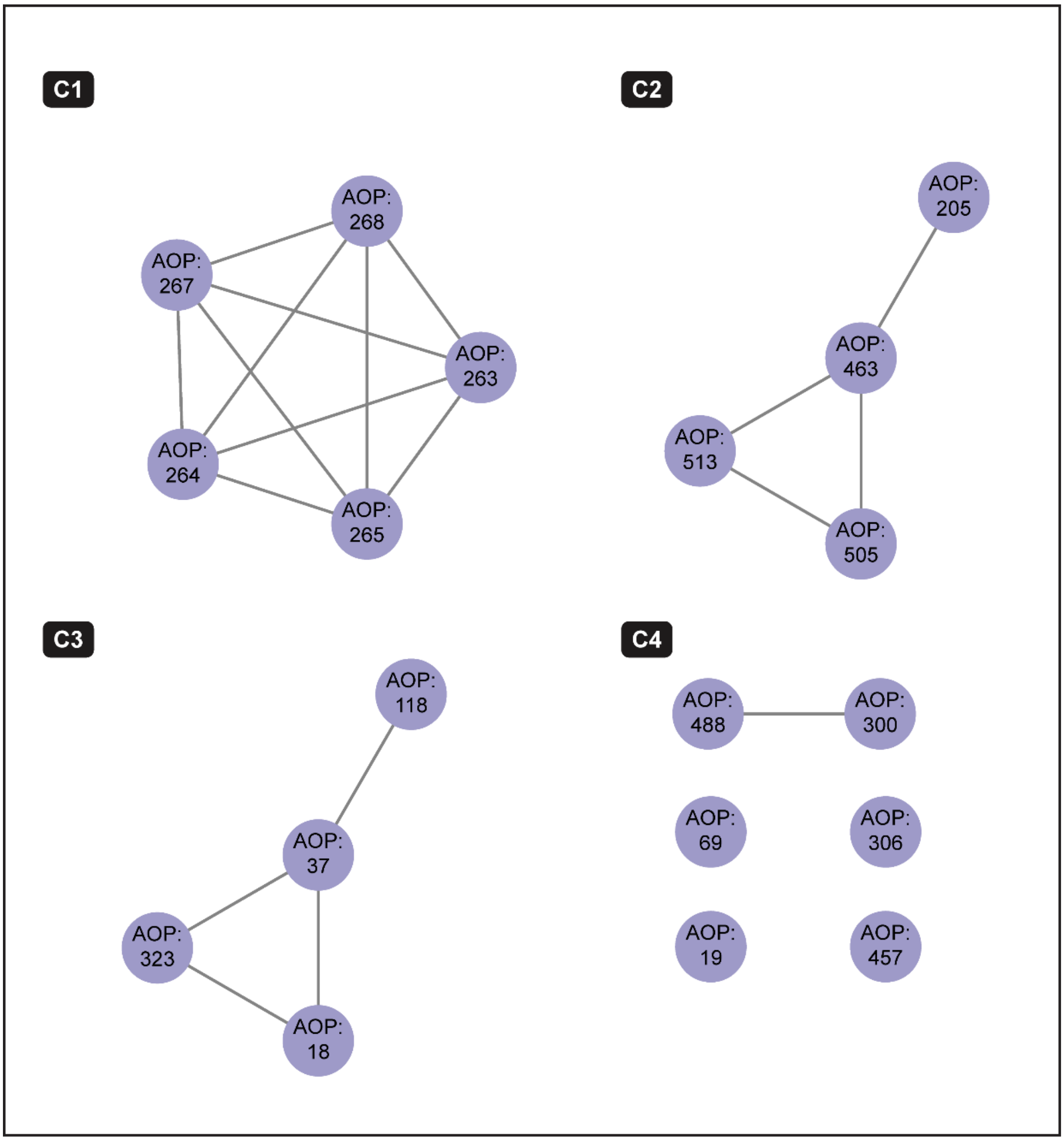

Fig. S10 Undirected network of Bis(2-ethylhexyl) phthalate (DEHP)-AOPs. Each node corresponds to DEHP-AOP and an edge between two nodes denotes that the two AOPs share at least one KE. This undirected network has 4 connected components (with two or more nodes) which are labeled as C1, C2, C3 and C4, and 4 isolated nodes.

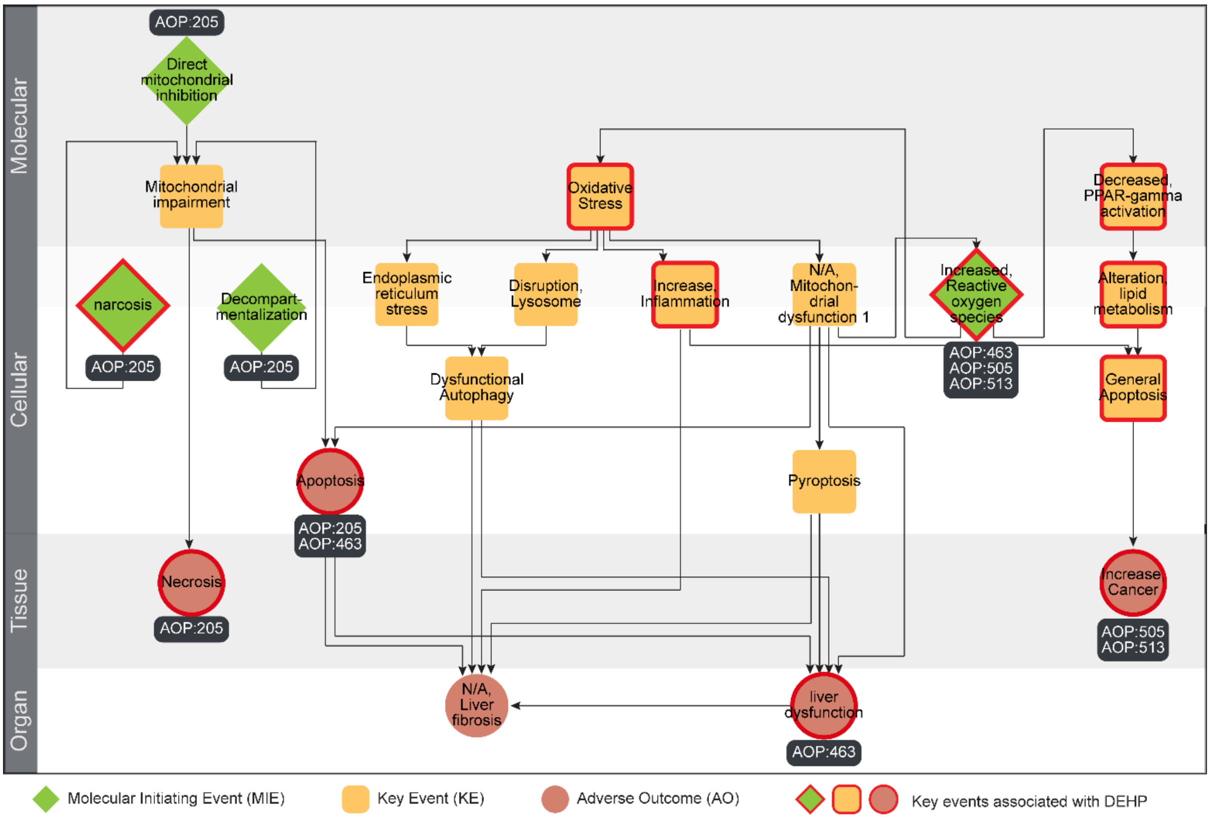

Fig. S11 Directed network corresponding to the connected component (C2) in the undirected DEHP-AOP network comprising 20 KEs and 29 KERs. Among the 20 KEs, 4 are categorized as MIEs (denoted as diamond), 5 are categorized as AOs (denoted as circle), and the remaining 11 are categorized as KEs (denoted as rounded square). The 11 KEs (including MIEs and AOs) associated with DEHP are marked in 'red'. In this figure, the 20 KEs are arranged vertically according to their level of biological organization.

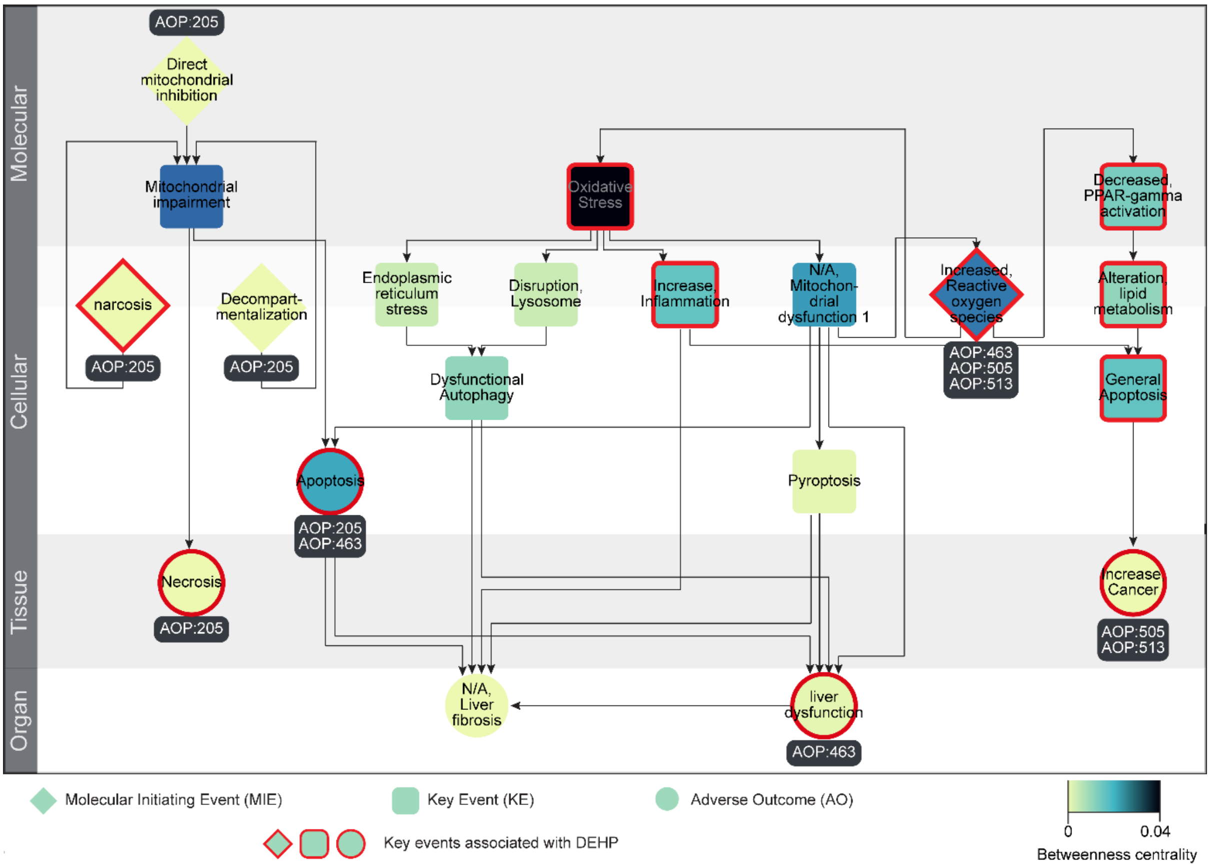

Fig. S12 Directed network corresponding to the connected component (C2) in the DEHP-AOP network, where the KEs (including MIEs and AOs) are colored based on their betweenness centrality values. The 11 KEs (including MIEs and AOs) associated with DEHP are marked in ‘red’. In this figure, the 20 KEs are arranged vertically according to their level of biological organization.

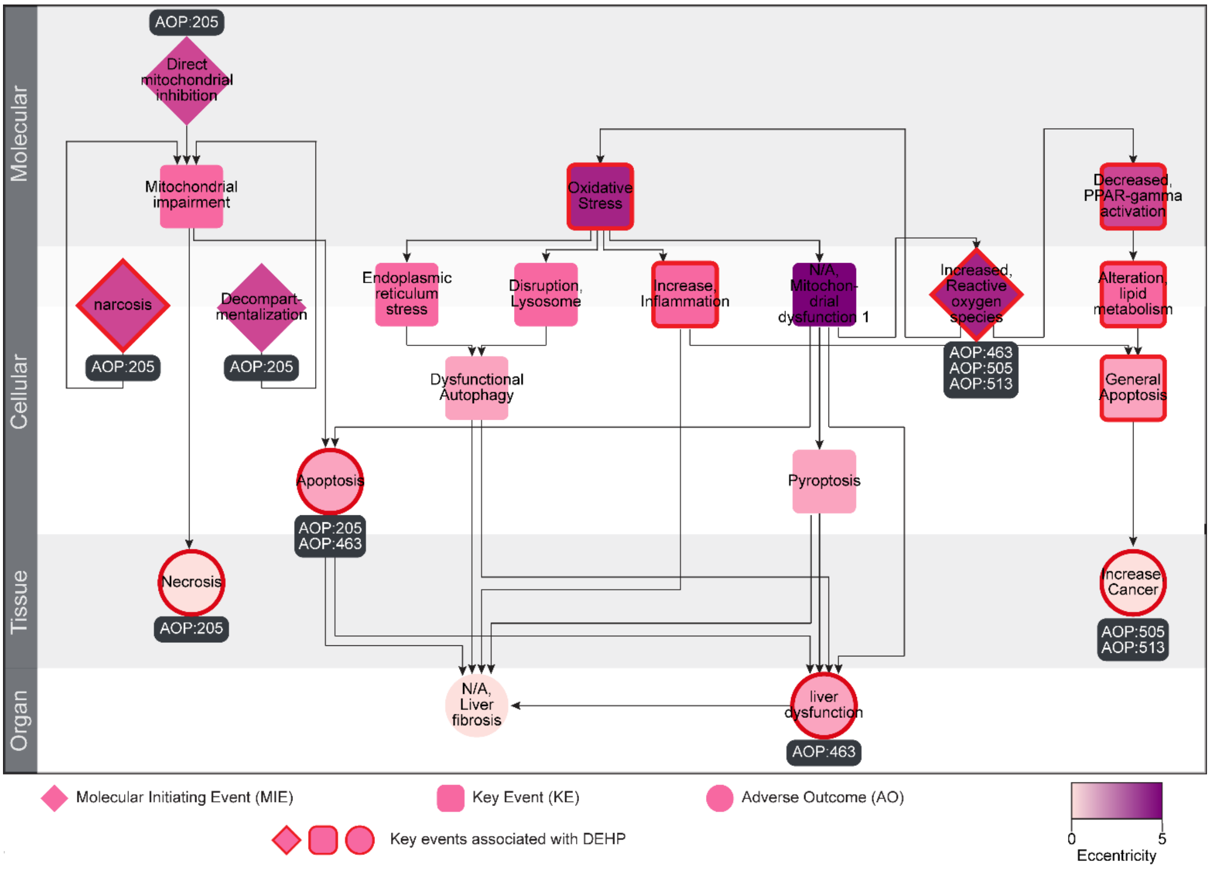

Fig. S13 Directed network corresponding to the connected component (C2) in the DEHP-AOP network, where the KEs (including MIEs and AOs) are colored based on their eccentricity values. The 11 KEs (including MIEs and AOs) associated with DEHP are marked in 'red'. In this figure, the 20 KEs are arranged vertically according to their level of biological organization.

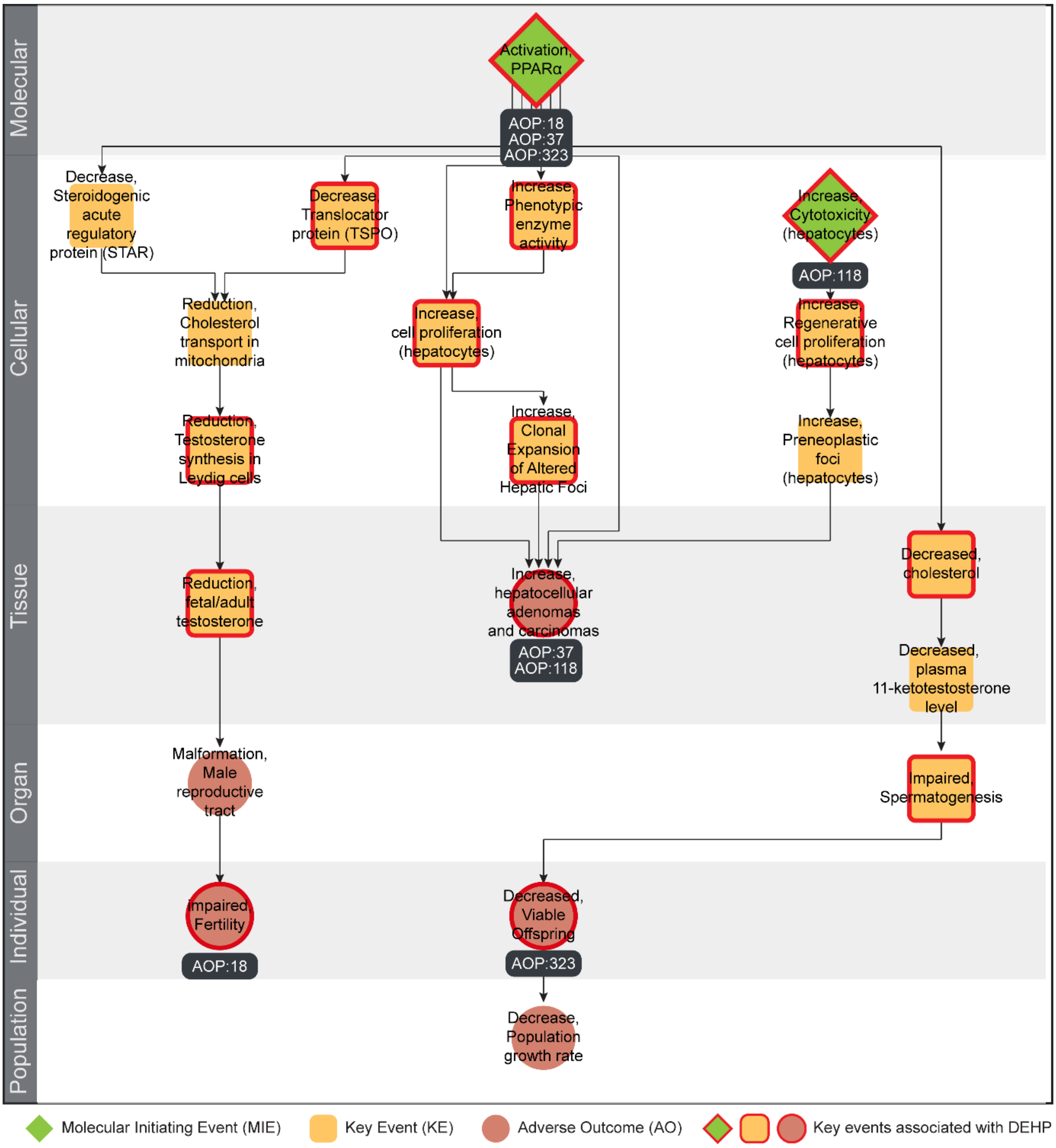

Fig. S14 Directed network corresponding to the connected component (C3) in the undirected DEHP-AOP network comprising 20 KEs and 23 KERs. Among the 20 KEs, 2 are categorized as MIEs (denoted as diamond), 5 are categorized as AOs (denoted as circle), and the remaining 13 are categorized as KEs (denoted as rounded square). The 14 KEs (including MIEs and AOs) associated with DEHP are marked in 'red'. In this figure, the 20 KEs are arranged vertically according to their level of biological organization.

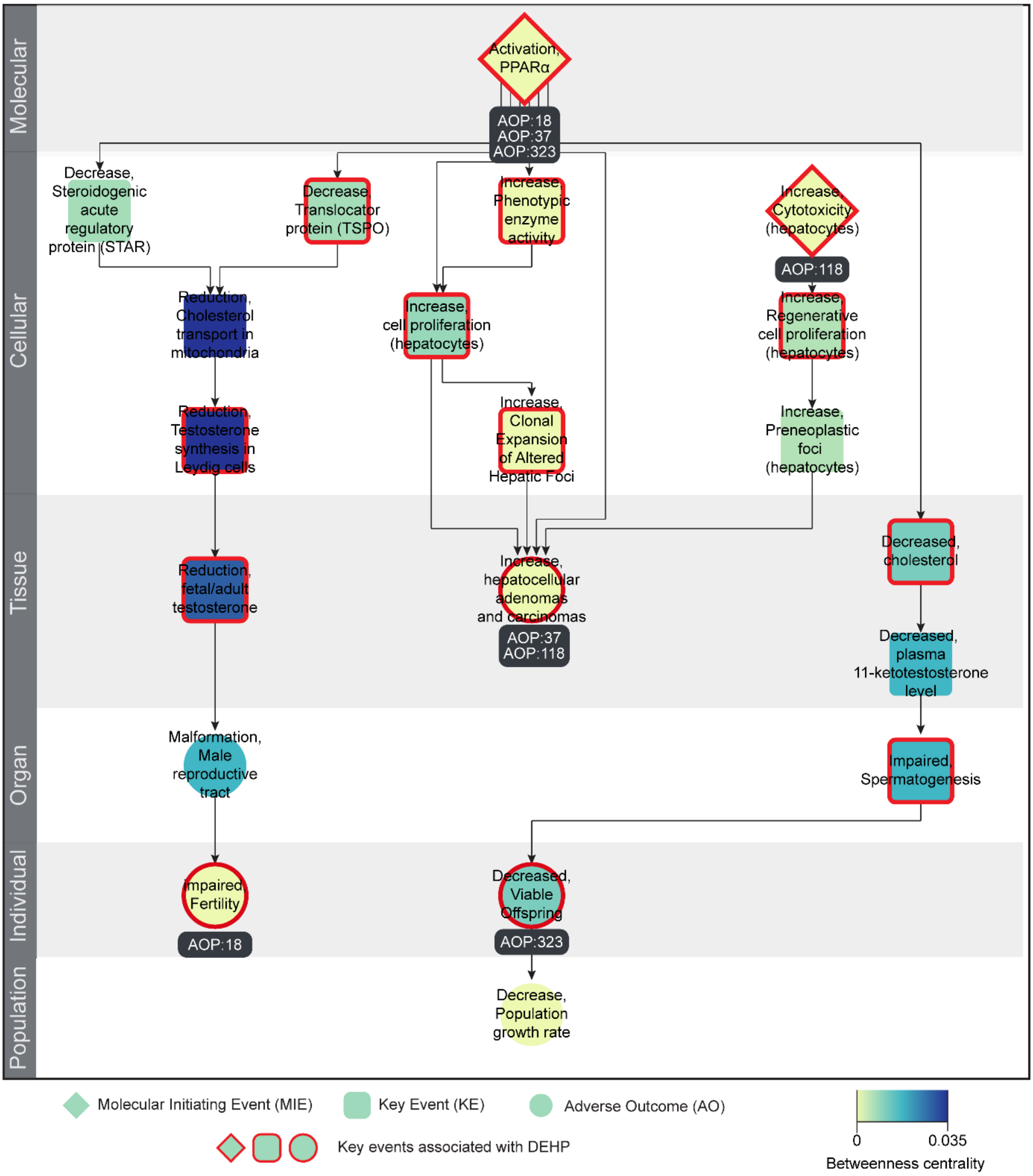

Fig. S15 Directed network corresponding to the connected component (C3) in the DEHP-AOP network, where the KEs (including MIEs and AOs) are colored based on their betweenness centrality values. The 14 KEs (including MIEs and AOs) associated with DEHP are marked in 'red'. In this figure, the 20 KEs are arranged vertically according to their level of biological organization.

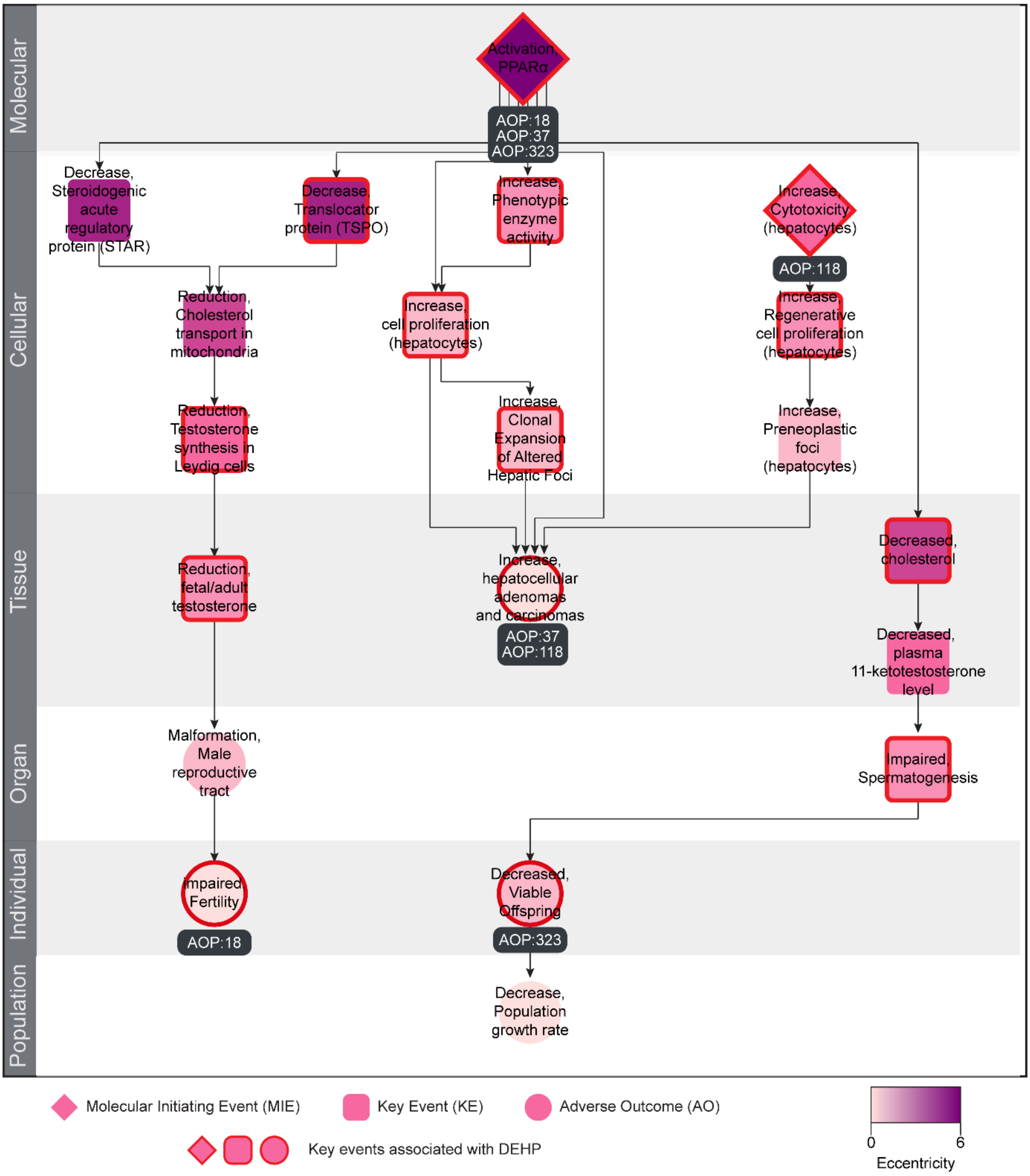

Fig. S16 Directed network corresponding to the connected component (C3) in the DEHP-AOP network, where the KEs (including MIEs and AOs) are colored based on their eccentricity values. The 14 KEs (including MIEs and AOs) associated with DEHP are marked in 'red'. In this figure, the 20 KEs are arranged vertically according to their level of biological organization.
